# Hydrological prediction in ungauged basins based on spatiotemporal characteristics

**DOI:** 10.1371/journal.pone.0313535

**Published:** 2025-01-10

**Authors:** Qun Zhao, Yuelong Zhu, Yanfeng Shi, Rui Li, Xiangtian Zheng, Xudong Zhou

**Affiliations:** 1 School of Computer Engineering, Nanjing Institute of Technology, Nanjing, Jiangsu, China; 2 College of Computer and Information, Hohai University, Nanjing, Jiangsu, China; 3 Institute of Ocean Engineering, Ningbo University, Ningbo, Zhejiang, China; 4 School of Civil and Environmental Engineering, Ningbo University, Ningbo, Zhejiang, China; Korea University, KOREA, REPUBLIC OF

## Abstract

Hydrological prediction in ungauged basins often relies on the parameter transplant method, which incurs high labor costs due to its dependence on expert input. To address these issues, we propose a novel hydrological prediction model named STH-Trans, which leverages multiple spatiotemporal views to enhance its predictive capabilities. Firstly, we utilize existing geographic and topographic indicators to identify and select watersheds that exhibit similarities. Subsequently, we establish an initial regression model using the TrAdaBoost algorithm based on the hydrologic data from the selected watershed stations. Finally, we refine the initial model by incorporating multiple spatiotemporal views, employing semi-supervised learning to create the STH-Trans model. The results of our experiments underscore the efficiency of the STH-Trans model in predicting runoff for ungauged basins. This innovation leads to a substantial increase in model accuracy ranging from 7.9% to 30% compared to various conventional methods. The model not only offers data support for water resource management, flood mitigation, and disaster relief efforts, but also provides decision support for hydrologists.

## 1 Introduction

In recent years, the advancement of the big data era and machine learning has significantly improved the accuracy of hydrological prediction. However, while most forecasting models excel in predicting watershed hydrology using rich monitoring station data, accurate hydrological forecasting for ungauged areas remains challenging. Despite the relatively complete network of current hydrological stations, remote watersheds are still lacking monitoring stations or with limited data from newly established stations (observation data less than six months) [1]. These remote and underserved areas pose a significant threat to residents’ property and life safety, particularly during continuous rainstorms leading to potential flood events.

For the ungauged basins with sparse data, the traditional hydrological model cannot be effectively trained and predicted. A hydrological data-driven model relies on a large amount of data information which rules are extracted from and then predicted. Therefore, the lack of a large number of historical data makes data-driven models useless for ungauged areas. In hydrology, the usual method is to find the nearest basin stations for parameter transplantation and build the correlation through modeling [2]. These methods usually require further process understanding and estimation methods and comprehensive knowledge across processes and regions [3]. At present, there are the following problems with hydrological prediction in ungauged areas [4]:

**1. Low universality**. The existing methods not only require a large number of parameter calculations [5], but also can only perform parameter migration for specific watersheds [6]. It cannot be applied to multiple watersheds and lacks universality.

**2. Strong professional dependence**. The migration method of hydrological parameters depends on the physical hydrological process [7], which requires professional knowledge and methods, as well as cross process, regional comprehensive experience to establish the model.

**3. Low prediction accuracy** [8]. The method of transplanting the parameters of the nearest station to the target station depends on the similarity between watersheds. If the hydrological conditions of the two basins are not so similar, they need to be slowly corrected according to the actual situation.

**Fig 1:**
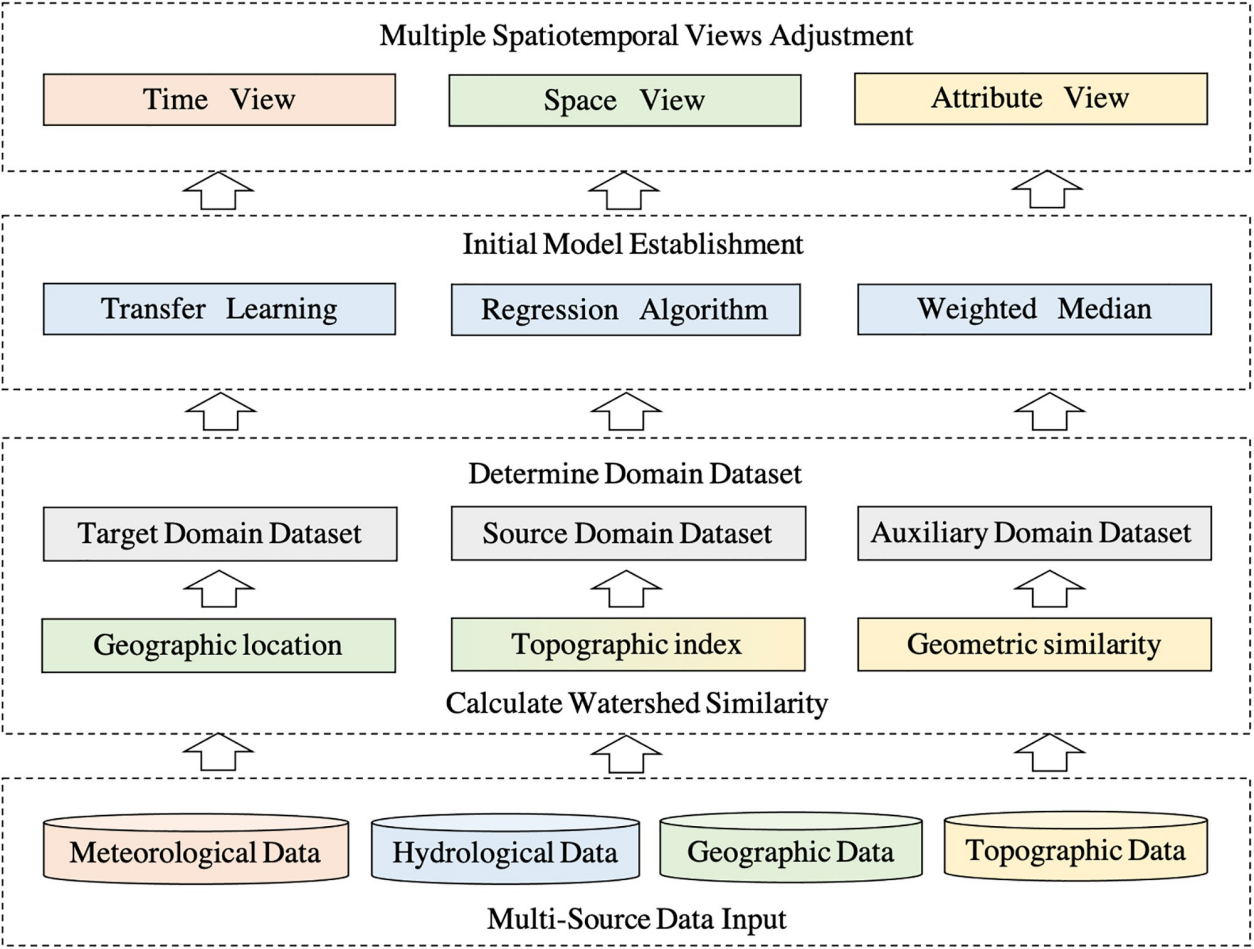
Framework of STH-Trans model, from the multi-source data as fundamental input (bottom of the entire framework) to the spatiotemporal views adjustment (top of the framework).

To solve the above problems, this paper proposes a hydrological prediction method for ungauged basins based on transfer learning and uses multiple space-time perspectives for adjustment, as shown in Fig 1. The model proposed in this paper not only improves the accuracy of hydrological transport, but also reduces reliance on expert knowledge, and improves the efficiency and universality of hydrological prediction.

The specific framework process is as follows:

1. Multi-Source Data Input: The bottom layer of the framework is the basic input of the model. The input data includes hydrological data, meteorological data, geographic location information, and terrain attributes.
2. Calculate Watershed Similarity: This is to determine the domain dataset. First, calculate the geographical distance between each hydrological station and the target point, and then select the stations within a certain distance to calculate the geometric similarity, terrain similarity and geographical location relationship for comprehensive judgment.
3. Determine Domain Dataset: Based on domain similarity, determine the target domain dataset, source domain dataset, and auxiliary domain dataset to participate in training.
4. Initial Model Establishment: The model improves the Transfer Learning algorithm and applies it to the regression task. At the end of the algorithm, the regression results are obtained by taking the median of the weighted basis learner.
5. Multiple Spatiotemporal Views Adjustment: Train three regressors starting from the time, spatial, and attribute views. Continuously adjust the average and difference values of any two regressors to obtain and output the final predicted value.

## 2 Related works

Predictions in Ungauged Basins (PUB), as the second International Hydrological Decade, was officially launched in 2003. Hrachowitz M et al. [9] reviews the work that has been done under the six science themes of the PUB Decade and outlines the challenges ahead for the hydrological sciences community. Tim et al. [10] took an ungauged area in southern Cambodia as an example, coupled the rainfall runoff model with the irrigation reservoir, used remote sensing data to drive, and professional knowledge to constrain parameters to establish a hydrological prediction model. Kong et al. [11] proposed a physical process driven distributed model, TOPKAPI model, to forecast floods in areas without data through parameter transplantation. Lance et al. [12] used the artificial neural network model to predict the runoff in ungauged areas and took the time lag records of precipitation and temperature as the input of the model. The experimental results show that the hourly flow prediction is better than the daily flow prediction. Tara et al. [13] proposed a multi modeling research method, using four regional models (two data-driven models and two hydrological models) for continuous daily flow estimation, and combining the four models to improve the model performance. Yu et al. [14] proposed an improved hydrological prediction model based on empirical model, radial basis function and autoregressive model for forward prediction of runoff series. Oruche R et al. [15] study the methodology behind Transfer Learning through fine-tuning and parameter transferring for better generalization performance of streamflow prediction in data-sparse regions. Chu KyungSu et al. [16] used the support vector machine method, the random forest method and the XGBoost. The threshold rainfall of the ungauged watersheds was calculated using the XGBoost technique and verified through past rainfall events and damage cases. Rasheed Zimeena et al. [17] presents a prototype ML-based framework for flood warning and flood peak prediction on the recent success of Machine Learning models on streamflow prediction. Choi Jeonghyeon et al. [18] incorporates vegetation modules into hydrological models to effectively improve accuracy. Demirel et al. [19] found that adding spatial pattern evaluation to the traditional temporal evaluation of hydrological models can assist in identifying optimal parameter sets. Swilla Livingstone et al. [20] aims to calibrate and verify the model for runoff prediction of ungauged basin using the other river flow data. The sub-catchment geospatial characteristics were delineated using the Digital Elevation Model of the study area in ArcGIS to help improve model’s performance. Giudicianni C. [21] discusses the impact of different rainfall events on ungauged basins, and also proves that basins are influenced by terrain, area, and rainfall.

In summary, machine learning method provides new ideas for hydrological prediction in ungauged basins. The spatial patterns, including vegetation coverage, watershed area and others, can also improve the accuracy of hydrological prediction in ungauged basins. Although more and more studies consider using machine learning to solve the PUB problem, there are still some problems, including low prediction accuracy and weak universality. Moreover, there are still no stations or historical data available for many small and medium-sized rivers. How to combine machine learning, transfer learning, and spatial features in the era of big data to predict the regions with sparse hydrological data and provide a model with good prediction accuracy while reducing the dependence on expert knowledge has become a complex problem.

## 3 Domain dataset selection based on spatial similarity

### 3.1 Basic definition

Transfer learning [22–25] is to mine new knowledge on the basis of existing knowledge and find the relationship between existing knowledge and new knowledge. The basic definitions are as follows:

**Domain** (*D*): *D* = {*X*, *P*(*X*)}, It is composed of data characteristics and data distribution. Generally speaking, it can be understood as a specific field at a certain time.

**Task** (*T*): *T* = {*Y*, *f*(⋅)}, It is composed of objective function and learning result. Generally speaking, it can be understood as something to do.

**Source Domain** (*D*_*S*_): *D*_*S*_ = {(*x*_*s*1_, *y*_*s*1_), ⋯, (*x*_*sn*_, *y*_*sn*_)}, *x*_*si*_ ∈ *X*_*S*_, *y*_*si*_ ∈ *Y*_*S*_. A domain with existing knowledge. Modeling requires the use of knowledge from the Source Domain.

**Target Domain** (*D*_*T*_): *D*_*T*_ = {(*x*_*T*1_, *y*_*T*1_), ⋯, (*x*_*Tn*_, *y*_*Tn*_)}, *x*_*Ti*_ ∈ *X*_*T*_, *y*_*Ti*_ ∈ *Y*_*T*_. A domain without knowledge and requiring knowledge learning tasks.

Transfer learning is based on source domain knowledge [26]. By reducing the data distribution difference between source domain and target domain, knowledge transfer is achieved.

The purpose of hydrological forecast is to predict flood events, reduce the impact of disasters, and conduct reasonable reservoir operation. So, we will forecast runoff. There are many factors that affect the runoff. In addition to hydrological factors, it is also affected by geographical and topographic factors and meteorological factors, such as watershed area, watershed length, elevation, slope and rainfall.

The Transfer learning model needs source domain and target domain for training. Therefore, it is very important to select the appropriate source basin data for the model training. In addition, for ungauged basins, we need to find a basin that can replace the training of the target basin. This paper selects and marks the basin that is most similar to the target basin, and defines it as an auxiliary basin to replace the target basin for training. The specific data set is defined as follows:

**Target Basin(*t*)**: the area without hydrological data that needs to be relocated.

**Source Basin(*s*)**: a basin with hydrological data, which is used to establish a model and assist the target basin in prediction.

**Auxiliary Basin(*h*)**: *h* ∈ *s*, the basin most similar to the target basin.

The rainfall data of *t* is *P*_*t*_, the rainfall data of *h* is *P*_*h*_, the rainfall data of *s* is *P*_*s*_, the runoff data of *h* is *Q*_*h*_, the runoff data of *s* is *Q*_*s*_, the geographic data of *t* is *G*_*t*_, the geographic data of *h* is *G*_*h*_, the geographic data of *s* is *G*_*s*_, the terrain data of *t* is *T*_*t*_, the terrain data of *h* is *T*_*h*_ and the terrain data of *s* is *T*_*s*_.

### 3.2 Selection of source basin and auxiliary basin based on spatial similarity

Similarity is compared by calculating the distance between the features of different things. For the same hydrological process, if the size of the hydrological related elements at each watershed point is proportional and their change trend is similar, then the two hydrological phenomena are similar [27]. In the dynamic process, if the two dynamic phenomena are similar, geometric similarity, kinematic similarity and dynamic similarity shall be generally met. In the physical process of hydrology, the geometric similarity considers the shape information, basin area, basin length and so on. The movement similarity considers the runoff direction and velocity of the river. For dynamic similarity, gravity and pressure of each location shall be considered, including elevation and slope of the basin [28, 29].

Based on the geographical location characteristics, topographic and geomorphic characteristics of each station, this section constructs three hydrological spatial relationship maps: Geometric Similarity Graph, Topographic Index Graph, and Geographic Location Graph. There is no hydrological data record in ungauged areas, such as runoff and water level. So we do not consider hydrological time series factors when constructing hydrological similarity map.

When the two rivers are geographically close, the hydrological process will also be similar to some extent because of the rainfall process and geographical conditions [30]. Therefore, it is necessary to consider the distance of the measuring station in the basin that is similar to the target basin. The distance formula is as follows:

$$\begin{array}{c} de_{i,j}=2R*atan2\left(\sqrt{a},\sqrt{\left(1-a\right)}\right) \end{array}$$ (1)

Where *R* is the radius of the earth, *atan*2(*x*, *y*) is a function that returns the arctangent of the specified *x* and *y* coordinate values.

The calculation formula of *a* is as follows:

$$\begin{array}{c} a=sin^{2}\left(\frac{\Delta \theta }{2}\right)+cos\alpha_{i}\cdot cos\alpha_{j}\cdot sin^{2}\left(\frac{\Delta \alpha }{2}\right) \end{array}$$ (2)

Where Δ*θ* is the latitude difference between two geographical locations, *α*_*i*_ and *α*_*j*_ is the longitude of two geographical locations, Δ*θ* is the longitude difference. The angle here must be in radians, not numeric latitude or longitude.

#### 3.2.1 Geometric similarity graph

The hydrological basin shape information includes basin area, basin length, etc. Based on the shape information, a geometric similarity graph is constructed. The similarity relationship is calculated by the weighted difference distance of the shape attribute values between two stations. The formula of Geometric Similarity Graph is as follows:

$$\begin{array}{c} GS_{a,t}=\sum_{m=1}^{n}\delta_{m}\left|a^{m}-t^{m}\right|,\sum_{m=1}^{n}\delta_{m}=1 \end{array}$$ (3)

Where *GS*_*a*,*t*_ is the geometric similarity distance between point *a* and *t*, where *a* is the surrounding basin, point *t* is the target basin, *n* is the number of attributes in the shape information, *δ*_*m*_ is the weighting coefficient, *a*^*m*^ is the *m*th attribute value in the *a* watershed shape information. *t*^*m*^ is the *m*th attribute value in the *t* watershed shape information. The smaller *GS*_*a*,*t*_ value is, the more similar it is to the target watershed.

#### 3.2.2 Topographic index graph

Affected by the topography of the basin, hydrological phenomena shows different hydrological processes. The topography not only affects the runoff direction, but also relates to the specific geographical distribution of the cumulative water volume. Topographic index is the cumulative trend of runoff at a certain point in the basin. Watershed points with similar topographic indexes have similar hydrological processes in theory. The formula of Topographic Index Graph is as follows [31]:

$$\begin{array}{c} T=ln(\frac{\alpha }{tan\beta }) \end{array}$$ (4)

Where *α* represents the accumulated water volume at any point in the basin, *tanβ* represents the runoff trend caused by gravity.

#### 3.2.3 Geographic location graph

The basin is affected by gravity and inertial force. The different velocity of water flow leads to different situations of rapid rise and fall of flow. According to the elevation difference and gradient difference between the two watersheds, the Geographic Location Graph is constructed. The formula is as follows:

$$\begin{array}{c} GL_{a,t}=\vartheta_{1}E_{a,t}^{d}+\vartheta_{2}aveslope_{a,t}^{d} \end{array}$$ (5)

Where *GL*_*a*,*t*_ is the combination of elevation difference and gradient difference between *a* and *t*, where *a* is the surrounding basin, point *t* is the target basin, *ϑ*_1_ and *ϑ*_1_ are the coefficients, $E_{a,t}^{d}$ is the normalized altitude difference of basin *a* and basin *t*, $aveslope_{a,t}^{d}$ is the normalized average gradient difference of basin *a* and basin *t*. The smaller *GL*_*a*,*t*_ value is, the more similar it is to the target watershed.

For the selection of the source basin, first filter the stations according to the horizontal distance, and then sort them according to the geometric similarity relationship, terrain index and geographical location. We can obtain three sorted station tables. Select the top *n*% stations in the three tables. If the selected stations rank in the top *n*% in all three tables, the data of this station will be placed in the source basin data table.

For the selection of auxiliary basin, they are sorted according to the difference value of topographic index. Three basins with small topographic index difference are selected, and one of the most similar basins is selected as the auxiliary basin according to the geometric relationship and geographical location relationship. The runoff information from the auxiliary basin stations will be used to establish the initial hydrological prediction model.

## 4 STH-Trans model

### 4.1 H-Trans model based on transfer learning

There is no available hydrological station in the ungauged area, so the required information cannot be effectively mined. Therefore, based on the idea of transfer learning, this section takes the runoff information of the auxiliary basin as the training data set of the target basin. Tradaboost algorithm [32] is improved based on the idea of adaboost algorithm, which is a widely used sample-based transfer learning method. Tradaboost algorithm filters the source domain data, removes the data inconsistent with the target domain sample distribution, updates the weight vector through the Boosting method, increases the weight of training data useful for the source domain data, and reduces the weight of invalid data with inconsistent distribution.

Aiming at the specific problems in hydrological field, a hydrological prediction model based on transfer learning is proposed. The model performs regression training operation based on the idea of Tradaboost algorithm [33]. The model adjusts the weight vector based on the prediction error, and then obtains the regression results by taking the median or weighted average of the weighted basis learners [34]. When the output *Y*_*i*_ of sample *X*_*i*_ is incorrect in the regression process, its actual error value $E=|{\hat{Y}}_{i}-Y_{i}|$ may be arbitrarily large, ${\hat{Y}}_{i}$ is the real value of sample *X*_*i*_. Therefore, the error calculation in this algorithm needs to be normalized so that its range is [0, 1], and then the cumulative error is used to calculate the redistributed weight.

This paper selects *M*5 *model tree* [35] as the base learner. The *M*5 *model tree* algorithm replaces constants with linear regression functions at leaf nodes and combines piecewise linear models to handle nonlinear problems. Compared with traditional linear regression algorithms, it can automatically segment the input space and accurately predict nonlinear data. Compared with regression trees, it has faster computational efficiency and higher prediction accuracy. Compared with neural network methods, it has interpretability [36]. The steps are as follows:

1. **Dataset input**: Set the training dataset as *D*. The first *n* samples of training set *D* are the source basin sample data, and the last *m* samples are the target basin sample data.
2. **Parameter set**: Set the base learner *M*5 *model tree*. This paper selects *M*5 *model tree* [37] as the base learner. Set the basic parameters and initialize the weight vector *W*^1^, to ${\;W}_{i}^{1}=\frac{1}{n+m}(1\leq i\leq n+m)$.
3. **Training model**: Train the base learner *h*_*t*_ with training data sets and weight vectors.
  Calculate the maximum error on the target basin sample set in the training set: $E={max}_{n+1}^{n+m}|y_{i}-h_{t}(x_{i})|$.
  Calculate the relative error on the target basin sample set in the training set: $e_{i}^{t}=|y_{i}-h_{t}(x_{i})|/E$.
  Where *y*_*i*_ is the real data, *h*_*t*_ is the basic learner, and *x*_*i*_ is the input data. Calculate the error rate of the learner *h*_*t*_ on the target basin sample set: $\varepsilon_{t}=\sum_{i=n+1}^{n+m}e_{i}^{t}w_{i}^{t}$, When *ε*_*t*_ > 0.5, reset it to 0.5.
  Update weight vector:

$w_{i}^{t+1}=w_{i}^{t}\beta^{e_{i}^{t}}/Z_{t},1\leq i\leq n$

$w_{i}^{t+1}=w_{i}^{t}{\beta_{t}}^{{1-e}_{i}^{t}}/Z_{t},n+1\leq i\leq n+m$

  Where $\beta_{t}=\varepsilon_{t}/\left(1-\varepsilon_{t}\right)and\beta =1/(1+\sqrt{2lnn/N})$, *Z*_*t*_ is the normalization factor.
4. **Model output**: Build the final regressor: $H(x_{i})=\sum_{t=1}^{N}h^{\prime }\left(x_{i}\right)ln\left(\frac{1}{\beta_{t}}\right)$, Where *h*′(*x*) is the median of *β*_*t*_*h*_*t*_(*x*).
  When *ε*_*t*_ is greater than 0.5, reset *ε*_*t*_ to 0.5. Each round of iteration adjusts the weight vector according to *ε*_*t*_. The sample with a large *ε*_*t*_ will reduce the influence factor in the next iteration. After multiple iterations, the data with smaller error rate *ε*_*t*_ of the target domain will be given higher weight, while the weight of others will be reduced.

### 4.2 STH-Trans model based on multiple spatiotemporal views

The hydrological data distribution of the source basin may be very different from that of the target basin. Since there are no stations in the area, some existing geographic data and calculated rainfall data of the target basin cannot fully estimate its hydrological status. Therefore, this chapter improves H-Trans by using semi-supervised learning [37–39] based on bifurcation and combining the existing rainfall data and geographic terrain data of the target basin to adjust H-Trans model.

#### 4.2.1 Multiple spatiotemporal view

The formation of hydrological phenomena is not only reflected in the scope of time and space, but also related to the geographical attributes of hydrological entities. When the spatial attributes between hydrological entities are more similar, the change trend of hydrological process will be similar. This paper adjusts the transfer model from three aspects: time view, space view, and attribute view. The three views are described below.

1. Time View

Hydrologic data mostly change with time. In the hydrological process, the hydrological situation will be affected not only by its own hydrological factors, but also by meteorological factors. Meteorological elements promote the whole hydrological process of the basin, which makes the different basins have different confluence characteristics. Rainfall is an important factor of hydrological process change, and it will cause obvious changes in runoff and water level. Therefore, the rainfall in the meteorological data is selected as the time series feature of the time view to be added to the training. The dataset based on Time View is defined as follows:

$$\begin{array}{c} D_{T}=Q\cup P \end{array}$$ (6)

Where *Q* is the runoff of the measuring station, and *P* is the area rainfall data corresponding to the measuring station.

2. Space View

Whether the hydrological phenomena between the two points are similar is related to the distance between the two points. The river channel distance cannot be calculated in the ungauged area, so we choose the horizontal distance as the measurement. The longitude and latitude are selected as the spatial coordinates to calculate the Euclidean Distance, and the altitude is selected as the geographic data for collaborative assistance. The dataset based on Spatial View is defined as follows:

$$\begin{array}{c} D_{s}=Q\cup G \end{array}$$ (7)

Where *G*(*l*_*t*_) = {*d*(*l*_*t*_, *l*_*s*_), *g*(*l*_*t*_, *l*_*s*_)}, *l*_*t*_ is the measuring station of the target basin, *l*_*s*_ is the measuring station of the source basin, *d*(*l*_*t*_, *l*_*s*_) is the horizontal distance between the target basin and the source basin, *g*(*l*_*t*_, *l*_*s*_) is the elevation difference between the target basin and the source basin, *G*(*l*_*t*_) is the spatial distance feature of the target watershed.

3. Attribute View

The topography, landform, soil vegetation and other conditions of a basin will have a great impact on the generation and change of runoff in the basin. These indicators are decisive parameters for hydrological forecasting. Select drainage area, drainage length, slope, vegetation coverage, terrain and other data in geographic attributes as attribute data [21, 40]. The dataset based on Attribute View is defined as follows:

$$\begin{array}{c} D_{A}=Q\cup T \end{array}$$ (8)

Where *T*(*l*_*t*_) = {*cs*(*t*(*l*_*t*_), *t*(*l*_*s*_)), *t*′(*l*_*t*_)}, *l*_*t*_ is the measuring station of the target basin, *l*_*s*_ is the measuring station of the source basin, *t*(*l*) =< *l*.*a*_1_, *l*.*a*_2_, *l*.*a*_3_, *l*.*a*_4_ >, *l*.*a*_1_ is the normalized drainage area, *l*.*a*_2_ is the normalized drainage basin length, *l*.*a*_3_ is the normalized slope, *l*.*a*_4_ is the normalized vegetation coverage, *cs*(*t*(*l*_*t*_), *t*(*l*_*s*_)) is the cosine similarity of two vectors, *t*′(*l*) is the terrain index, *T*(*l*_*t*_) is the attribute feature of the target basin.

#### 4.2.2 STH-Trans model establishment

**Fig 2:**
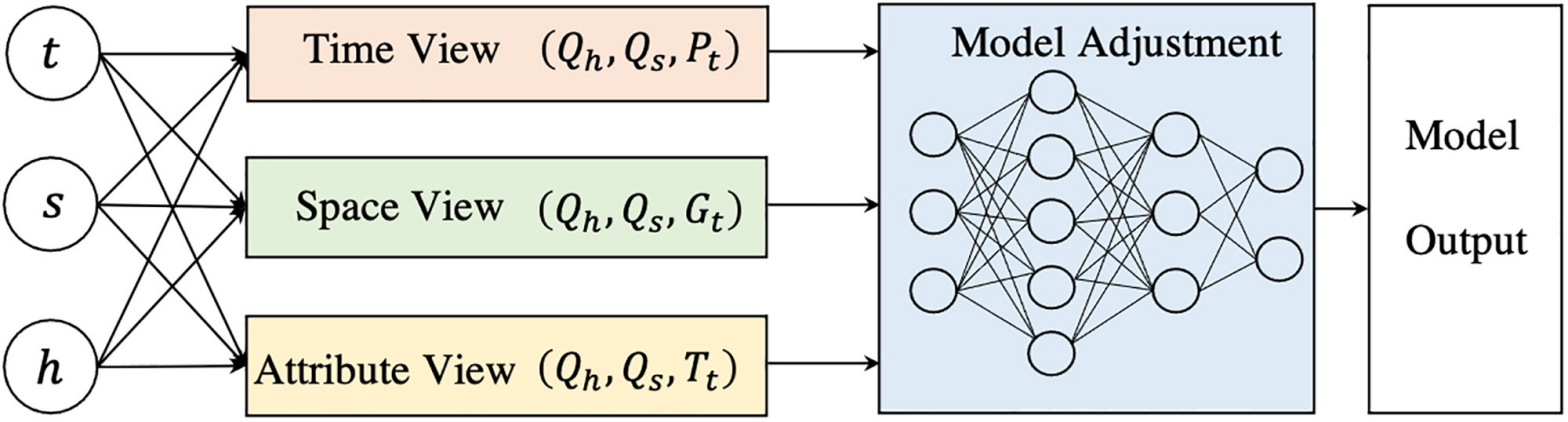
Multi spatiotemporal perspective adjustment.

This paper proposes a transfer learning algorithm based on the adjustment of multiple spatiotemporal views. Drawing on the idea of Tri-Training [41–45], starting from the three characteristic views of time, space and attribute, we use three regression functions based on these three views to determine how to select appropriate unlabeled samples for labeling, as shown in Fig 2.

Where *t* is the target watershed, *s* is the source watershed, *h* is the auxiliary watershed, *Q* is the runoff dataset, *P* is the rainfall dataset, *G* is the spatial dataset, and *T* is the attribute dataset.

The model first selects appropriate training data sets of source domain and target domain based on the idea of transfer learning and similarity analysis. Then start with the Time View, Space View and Attribute View, train three regressors based on these three views, and constantly adjust them through the difference value to finally get the final estimate.

The specific steps of model construction are as follows:

1. **Initial model construction**: Tradaboost regression algorithm is constructed according to the algorithm in Section 4.1, and the base learner is *M*5 *model tree*.
2. **Model adjustment**:
  (a) Use the data based on Time View, Space View and Attribute View and the hydrological data of source domain and auxiliary domain to establish three regressors for the target domain respectively *R*_1_, *R*_2_, *R*_3_.
  (b) Calculate the average and difference of the estimated values of any two regressors and put them into the empty sample set *L*.
  (c) Sort all samples in sample set *L*, and divide all sorted samples into *b* sub sample sets from top to bottom.
  (d) Select the sample of *a*% with the smallest difference in the sub sample set to participate in the calculation instead of the original sample set in the regression machine that did not participate in the calculation.
3. **Model output**: After the conditions are met or the iteration is completed, the average regression of the three regressors is output as the final result.

In the model adjustment step, if the difference between the estimated values of the other two regression functions is small, the average estimated values provided by them will obtain higher confidence. However, the size of the difference cannot be measured by a fixed value. For example, the estimated values of sample *A* and sample *B* are 10 and 100 respectively, obviously sample *A* is easier to obtain a smaller difference. Therefore, we sort the average of the estimated values in each iteration, and then partition the sorted samples from top to bottom. Find the samples with the smallest difference in the partition and add them to the next round of calculation until the iteration is completed.

## 5 Experimental analysis

### 5.1 Experimental dataset selection

**Fig 3:**
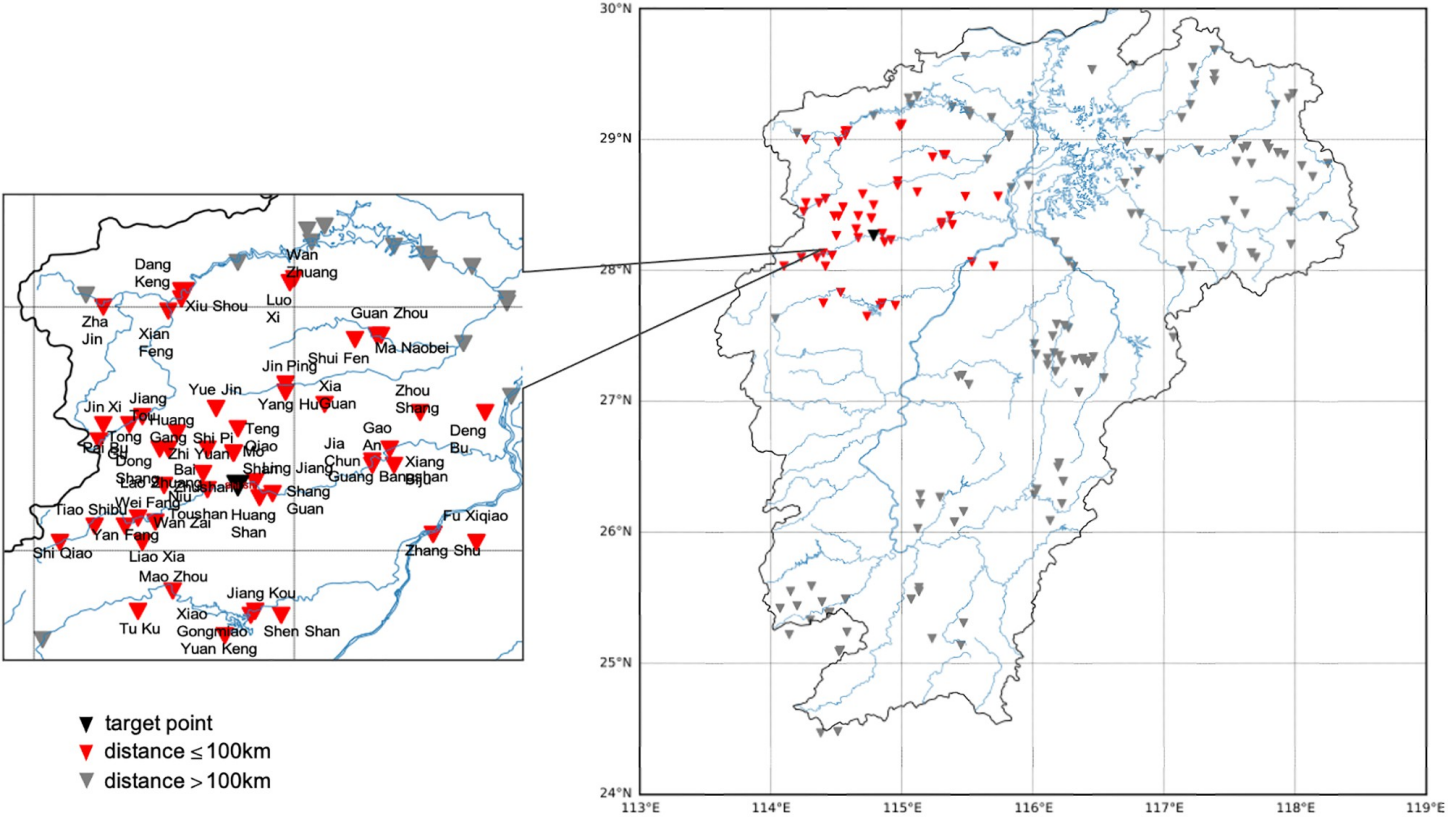
Distance from target watershed (Shi Shi as the example) to all selected stations. (The figure was generated by Python with basemap toolkit. The map source and license are accessible from https://github.com/matplotlib/basemap.).

The experiment uses data of hydrological stations in Jiangxi Province, China (see Fig 3). To verify the validity of the model, the experiment uses the basin with hydrological data as the ungauged area for prediction. All hydrological data of this station are only used for final comparison, not as training data in the experiment. Taking Shi Shi Station in Jiangxi Province (station code: 62312550, east longitude: 114.78, north latitude: 28.26) as an example, the station is located in Putian Village, Zhajin Town, Xiushui County, Jiangxi Province, and belongs to Xiushui River System. The drainage area of the station is 2807 *km*^2^, accounting for 73.3% of the Wanzai River basin area, belonging to the subtropical humid climate. The river section is basically straight and the channel is approximately trapezoidal. The width of the medium to high water channel is about 160 *m* to 230 *m*, and there are no tributaries upstream. The riverbed is composed of fine sand on the left bank, pebbles on the right bank, and some rocks. There are three sandbars located 400 *m* upstream of the section, growing weeds and trees. In some sections of the river below 306 *m* downstream, a floodplain appears on the left bank above 53.50 *m* water level. The water surface width has increased from over 200 *m* to about 300 *m*, and there is a left bend about 500 *m* downstream, with medium to high water playing a controlling role [46].

#### 5.1.1 Multiple spatiotemporal view

Select the source basin and auxiliary basin corresponding to the target basin. First, calculate the geographic distance according to the longitude and latitude coordinates, and then calculate the terrain similarity according to the distance. Because hydrological station measurements provide hydrological data, the longitude and latitude coordinates here use the coordinates of hydrological stations. The red triangles in Fig 3 refer to all stations within 100km from the target basin (indicated as the black triangle).

**Fig 4:**
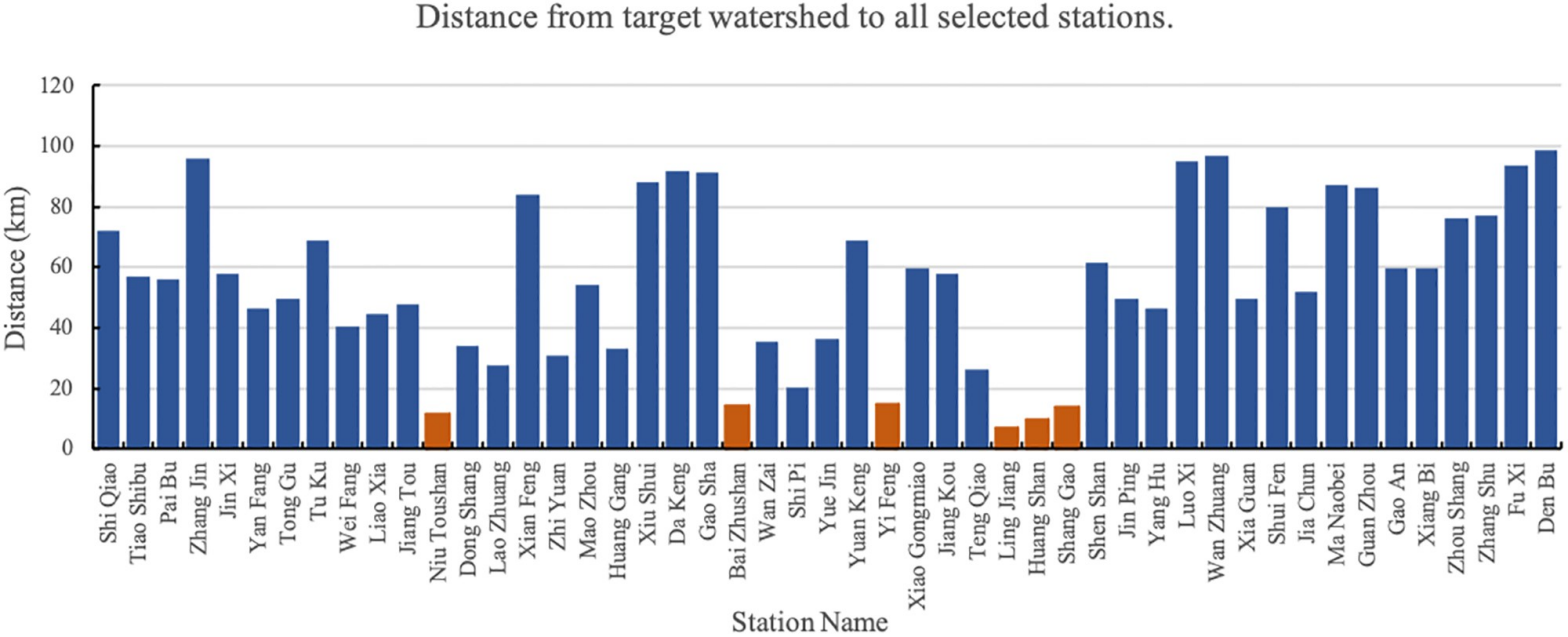
Distance from target watershed (Shi Shi as the example) to all selected stations.

The distance from the target basin (Shi Shi as the example) to all stations within 100 *km* is shown in Fig 4. In the figure, the orange bars represent stations with a distance of less than 15 *km*.

**Fig 5:**
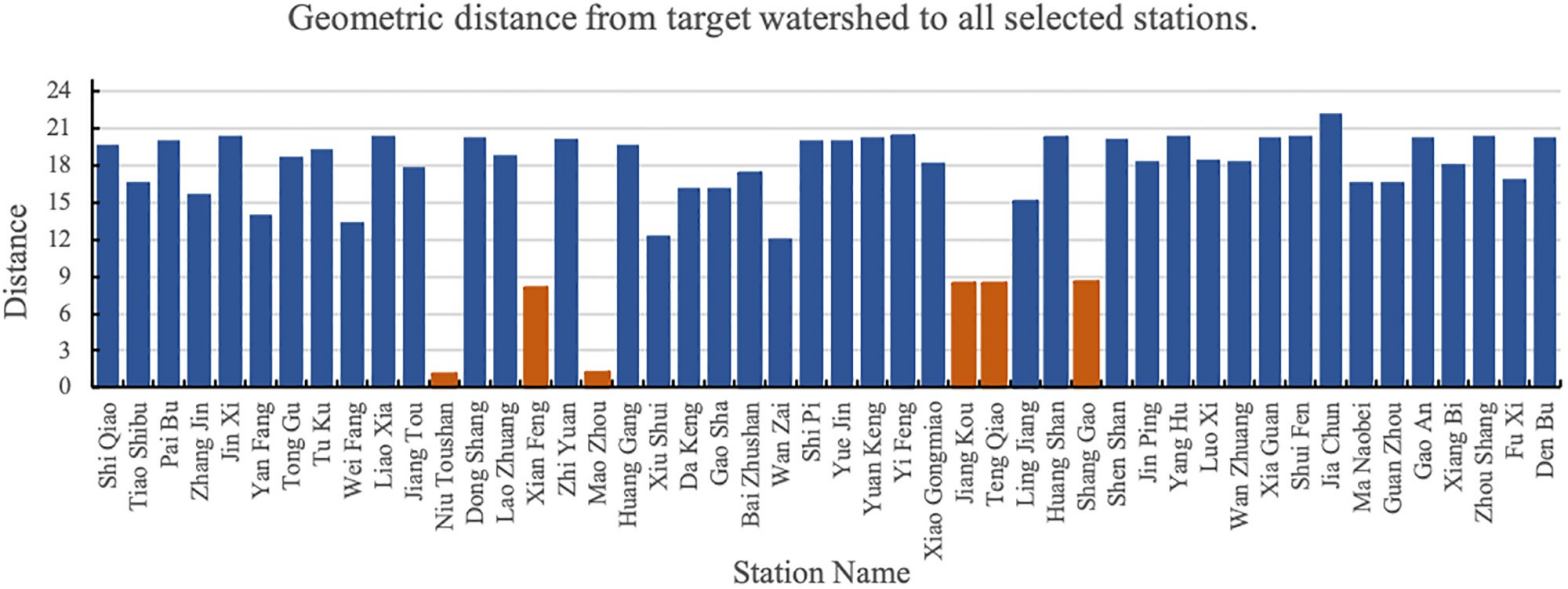
Geometric distance from target watershed (Shi Shi as the example) to all selected stations.

Next, we establish a geometric similarity graph, and calculate the geometric distance according to the shape information, watershed area, length, etc. The smaller the distance, the more similar it is. The geometric distances from target watershed to all selected stations are shown in Fig 5.

**Fig 6:**
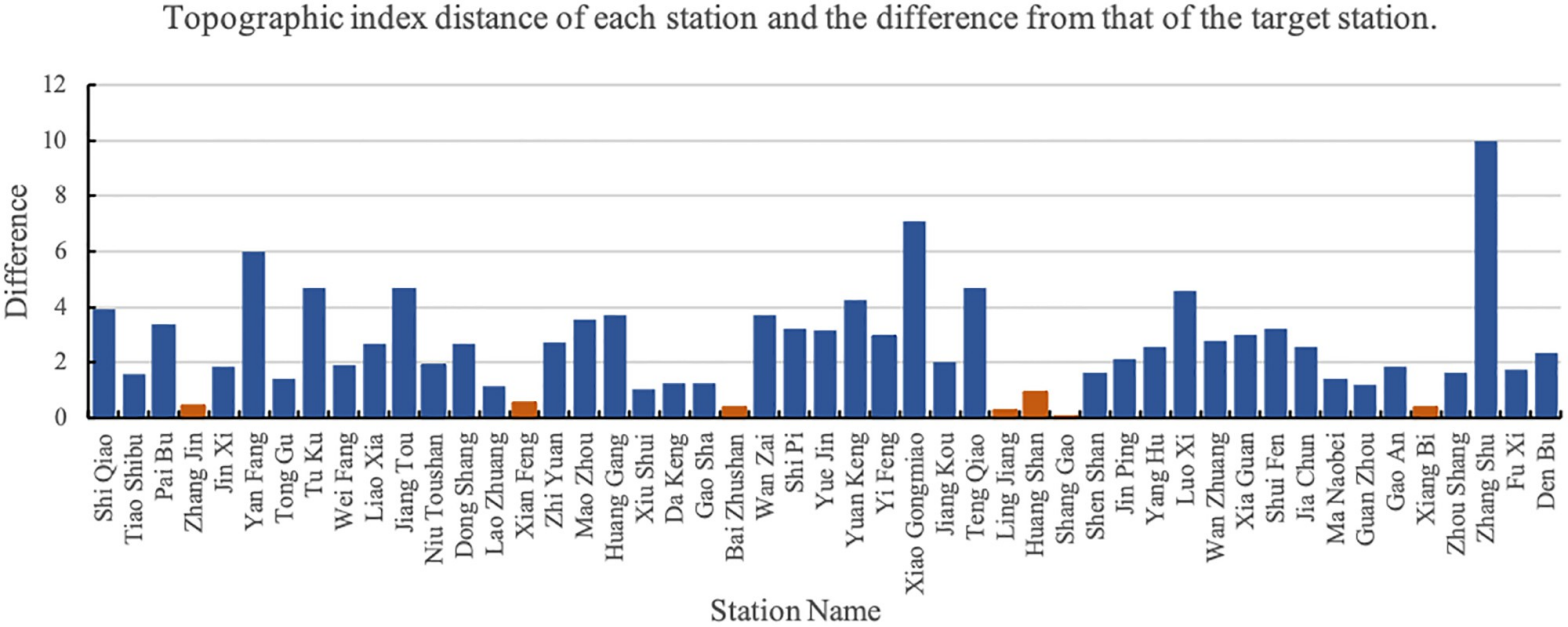
Topographic index distance between target watershed and all stations.

Then, we calculate the terrain index through ArcGIS. The terrain index of the target basin is 20.96. The closer the topographic index is to that of the target station, the more similar it is to the target basin. The terrain index is shown in Table 1 and the topographic index distance is shown in Fig 6:

**Table 1:** Station topographic index.

Shi Qiao	Tiao Shibu	Pai Bu	Zhang Jin	Jin Xi
24.87	22.52	24.35	21.37	19.12
Yan Fang	Tong Gu	Tu Ku	Wei Fang	Liao Xia
26.97	19.56	19.98	27.06	16.26
Jiang Tou	Niu Toushan	Dong Shang	Lao Zhuang	Xian Feng
25.63	22.86	23.63	19.81	20.42
Zhi Yuan	Mao Zhou	Huang Gang	Xiu Shui	Da Keng
23.71	24.51	24.68	21.97	22.22
Gao Sha	Bai Zhushan	Wan Zai	Shi Pi	Yue Jin
22.21	21.32	24.67	24.15	17.78
Yuan Keng	Yi Feng	Xiao Gongmiao	Jiang Kou	Teng Qiao
16.70	18.00	28.07	22.97	25.64
Ling Jiang	Huang Shan	Shang Gao	Shen Shan	Jin Ping
0.67	21.88	21.03	19.35	18.86
Yang Hu	Luo Xi	Wan Zhuang	Xia Guan	Shui Fen
18.42	25.53	18.18	17.98	17.75
Jia Chun	Ma Naobei	Guan Zhou	Gao An	Xiang Bi
18.42	22.35	19.74	22.81	21.33
Zhou Shang	Zhang Shu	Fu Xi	Den Bu	**Shi Shi**
19.34	30.92	22.68	18.63	**20.96**

In the figure, the orange bars represent stations with terrain index differences less than 1. The station with the smallest difference in terrain index is Shang Gao.

**Fig 7:**
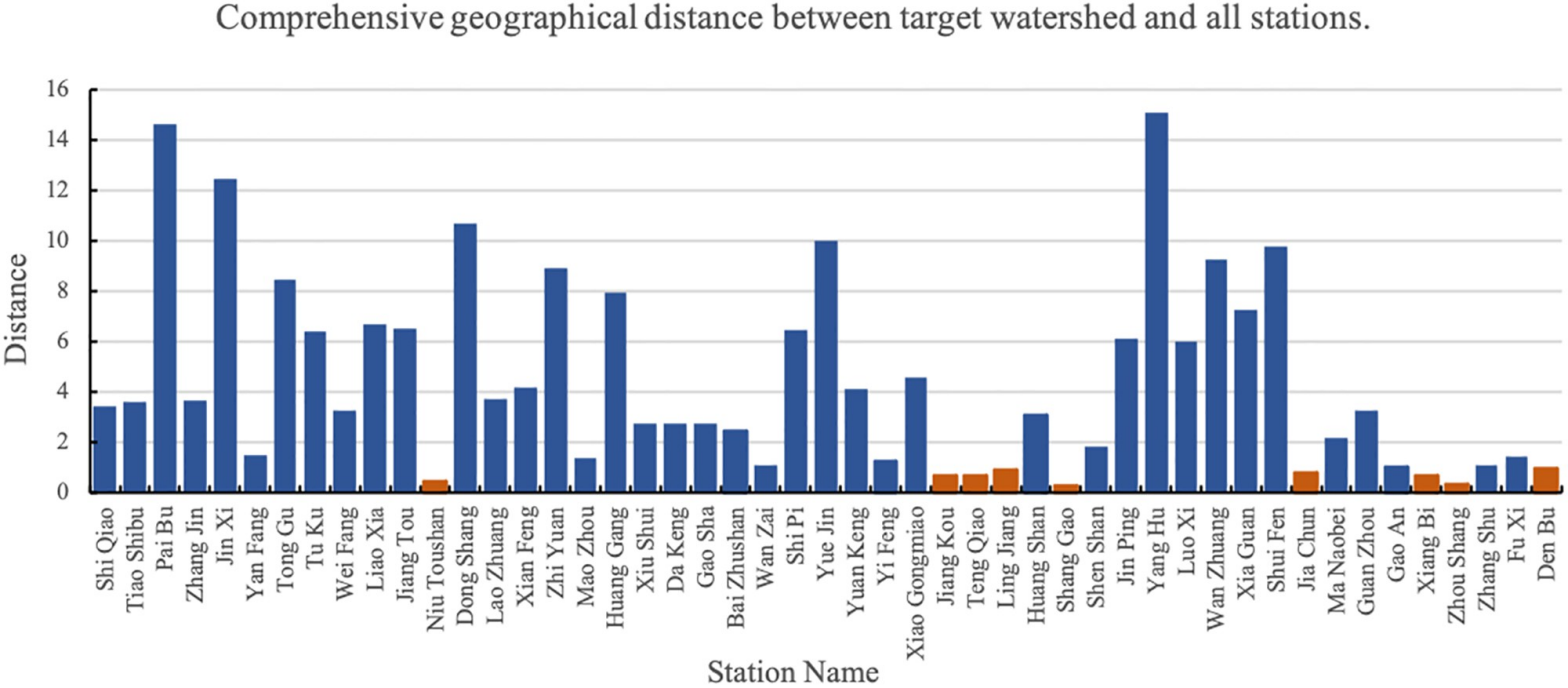
Comprehensive geographical distance between target watershed and all stations.

Finally, we establish a geographical location graph. Fig 7 shows the comprehensive geographic distance between the target watershed and all measurement stations. The smaller the value, the closer it is to the target basin.

In the figure, the orange bars represent stations with comprehensive geographic distances less than 1. Shang Gao is the station with the smallest difference.

**Fig 8:**
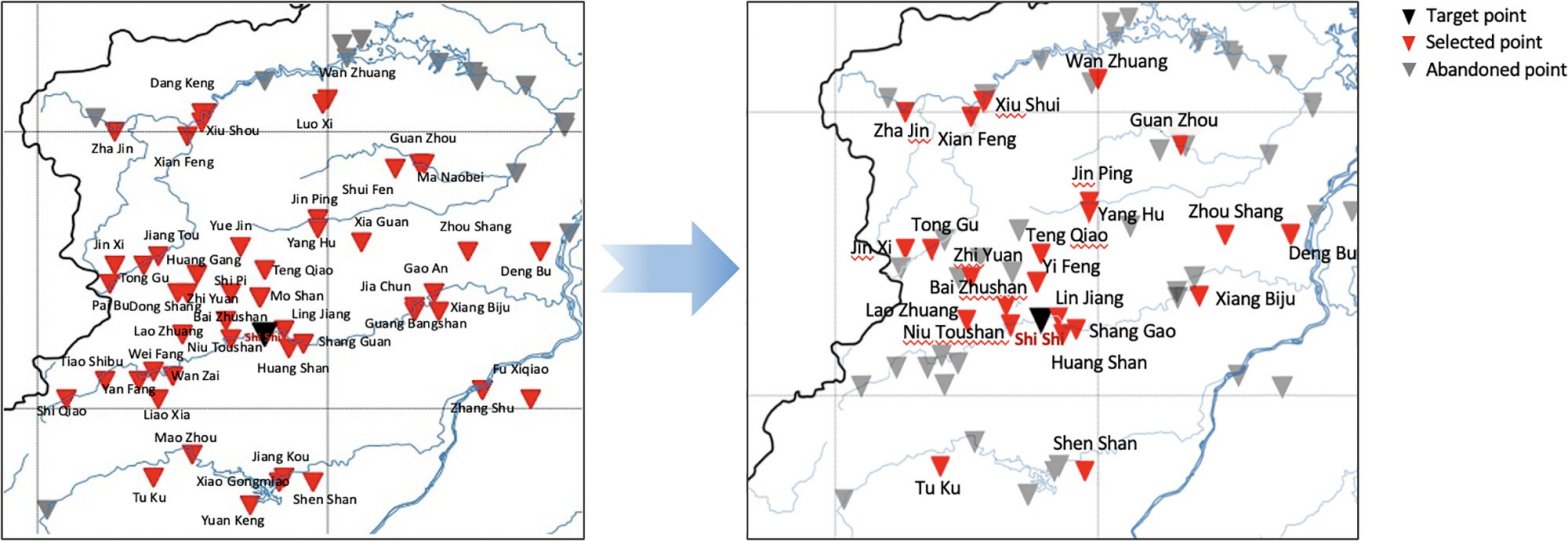
Selected source basins used in the transfer learning (in red triangles) after filtering out stations with missing data. (The figure was generated by Python with basemap toolkit. The map source and license are accessible from https://github.com/matplotlib/basemap.).

Through comprehensive comparison and removal of a large number of stations with missing data, 23 stations with small differences in terrain index and small geometric distance from the comprehensive geographic distance are selected. Among them, Shang Gao, Bai Zhushan and Ling Jiang are the basins with the smallest difference in topographic index. In consideration of its geometric distance and comprehensive geographical distance, Shang Gao, which has a relatively small gap, is selected as an auxiliary basin and directly put into the model to replace the target basin to help with migration and prediction. Other stations are put into the model as the source basins for transfer learning (see the red triangles shown in Fig 8).

**Fig 9:**
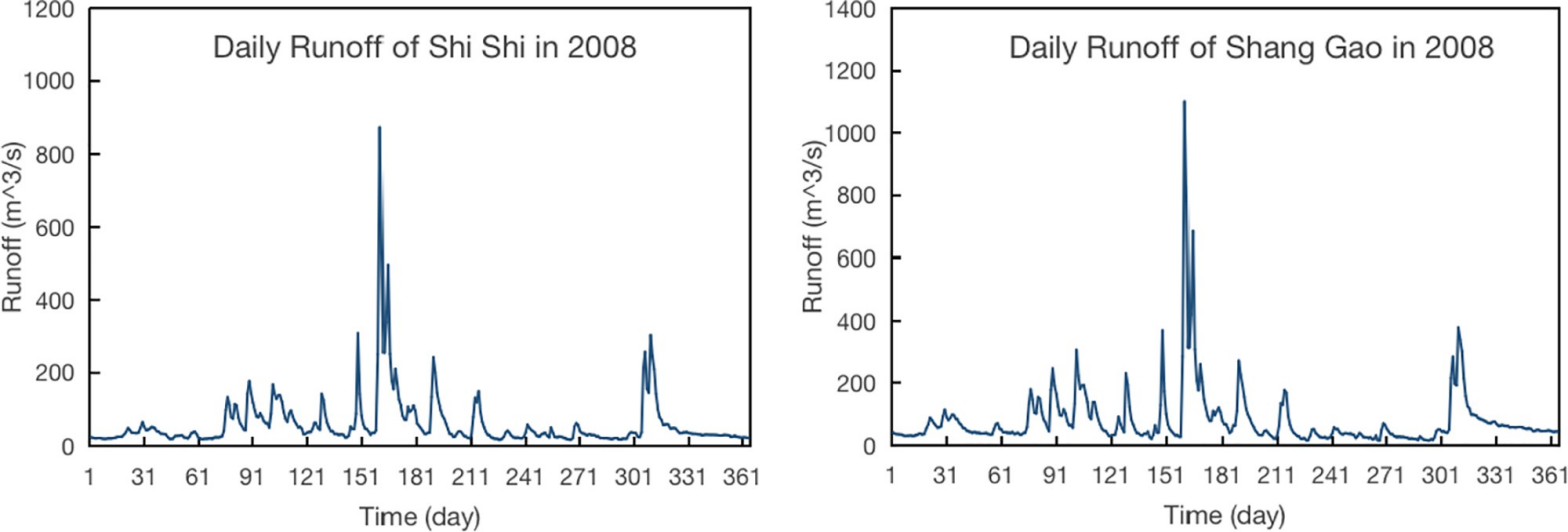
Daily runoff of Shi Shi and Shang Gao in 2008.

Based on the data analysis for the selected auxiliary basin, Fig 9 below shows the daily flows of Shang Gao and Shi Shi in 2008. It can be seen from the figure that although the runoff is different, the runoff change trend of the two watersheds is similar. Therefore, it is feasible for us to select the auxiliary basin to help the ungauged basin to predict.

#### 5.1.2 Selection of spatiotemporal data

**Fig 10:**
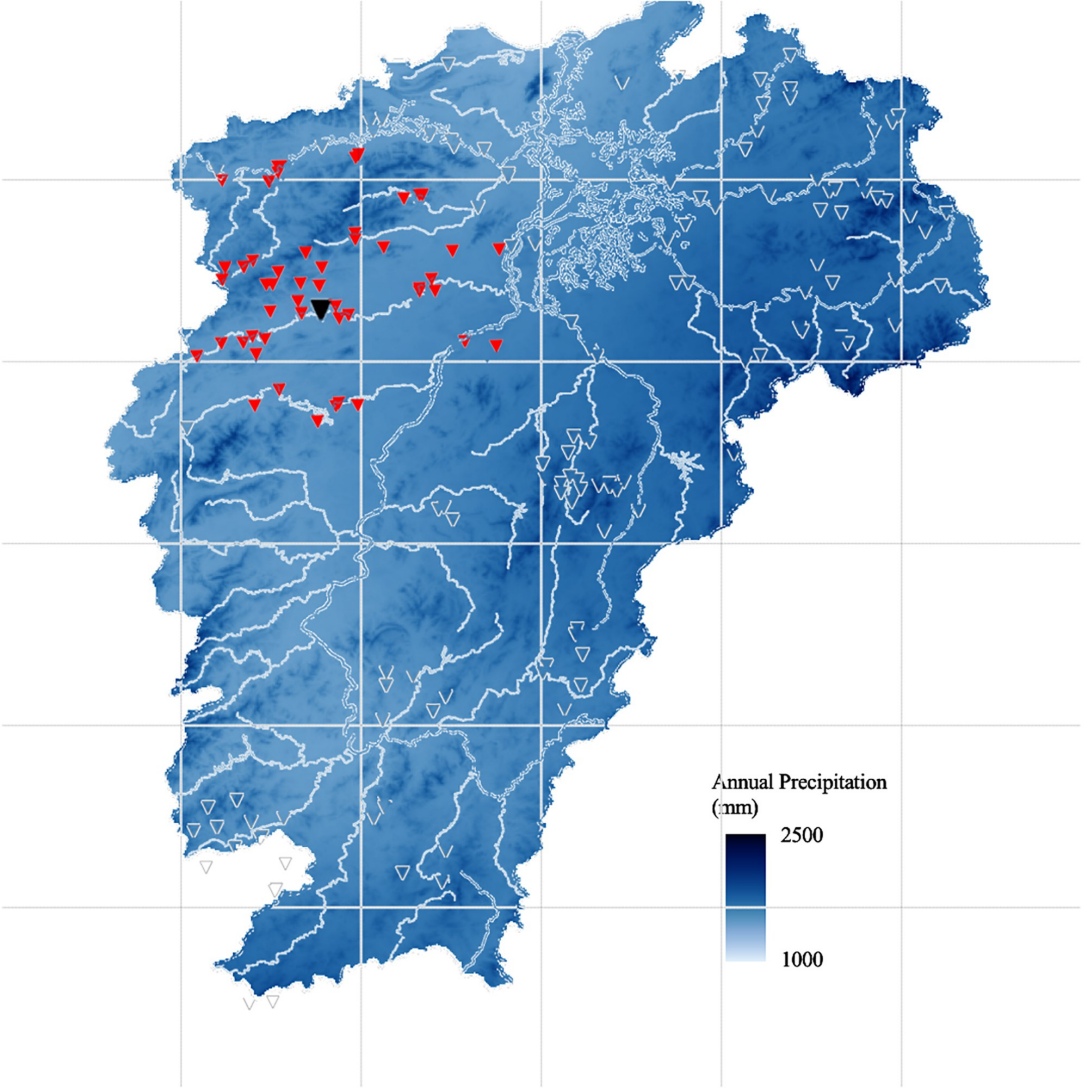
Annual rainfall map of Jiangxi Province. (The figure was generated by Python with basemap toolkit. The map source and license are accessible from https://github.com/matplotlib/basemap.).

*Time view dataset*. the five years runoff data from 2005 to 2009 of the source basin and auxiliary basin that have been normalized [47] are selected for the experiment, and there are 269,334 runoff data in total. In addition, the rainfall data of the target basin can be obtained by interpolating rainfall from the surrounding rainfall stations. The average rainfall of the first six hours and the current period of the source basin and the target basin are also input as time characteristics. However, because it is difficult to show hourly rainfall data for a period of time, we show the general distribution of annual rainfall in Fig 10. It is viewed that the rainfall is higher in the hill regions than that in the flat plains, which highlights the risk of flash floods.

**Fig 11:**
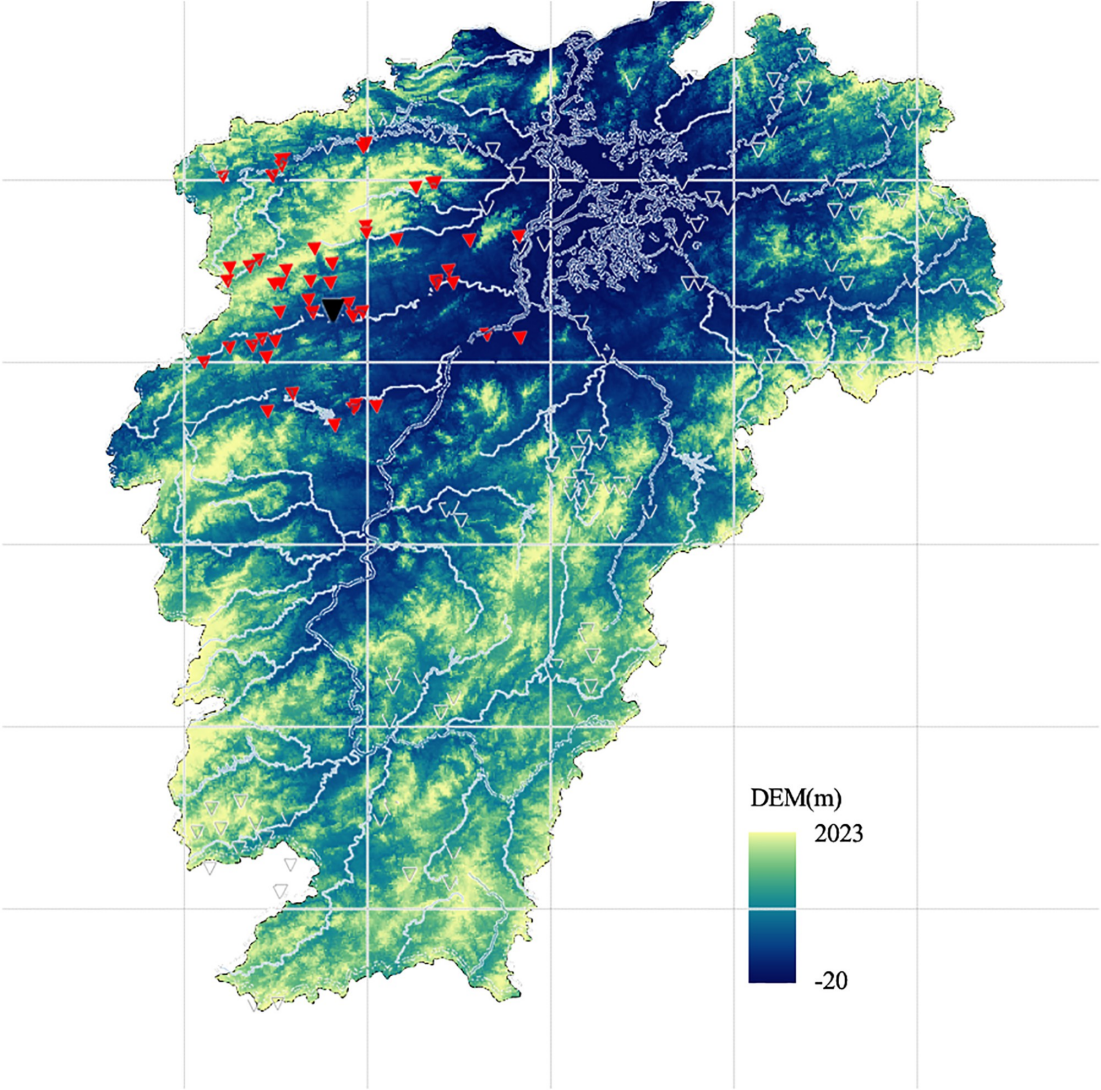
Digital elevation map of Jiangxi Province. (The red triangles refer to all stations within 100 *km* from the target basin (indicated as the black triangle). The figure was generated by Python with basemap toolkit. The map source and license are accessible from https://github.com/matplotlib/basemap.).

*Spatial view dataset*. spatial features are represented by spatial coordinates. The experiment includes 22 source basins, 1 auxiliary basin, and 1 target basin. Spatial features include longitude and latitude, elevation, and elevation difference of these basins, as shown in Table 2. The digital elevation map is shown in Fig 11.

**Table 2:** Altitude and distance between each station and target station.

Station	Shang Gao	Zha Jin	Jin Xi	Tong Gu
Distance(km)	13.59	95.98	57.75	49.39
Altitude(m)	48	159	332	305
Altitude Difference(m)	-8	103	276	249
Station	Lao Zhuang	Xian Feng	Bai Zhushan	Ling Jiang
Distance(km)	27.78	83.93	14.21	6.79
Altitude(m)	163	249	93	52
Altitude Difference(m)	107	193	37	-4
Station	Shen Shan	Jin Ping	Yang Hu	Guan Zhou
Distance(km)	61.59	49.73	46.29	86.20
Altitude(m)	99	236	298	135
Altitude Difference(m)	43	180	242	79
Station	Zhou Shang	Deng Bu	Teng Qiao	Yi Feng
Distance(km)	76.24	98.82	25.97	14.93
Altitude(m)	46	41	127	83
Altitude Difference(m)	-10	-15	71	27
Station	Zhi Yuan	Wan Zhuang	Xiu Shui	Niu Toushan
Distance(km)	31.01	96.96	87.93	11.59
Altitude(m)	271	371	182	75
Altitude Difference(m)	215	315	126	19
Station	Tu Ku	Huang Shan	Xiang Bi	
Distance(km)	68.76	9.89	59.53	
Altitude(m)	139	55	32	
Altitude Difference(m)	83	-1	-24	

*Attribute view dataset*. attribute feature data are obtained by mining from spatial data and using software such as ArcGIS, including terrain and geomorphic data such as watershed area (square kilometers), watershed length (kilometers), and vegetation coverage.

**Fig 12:**
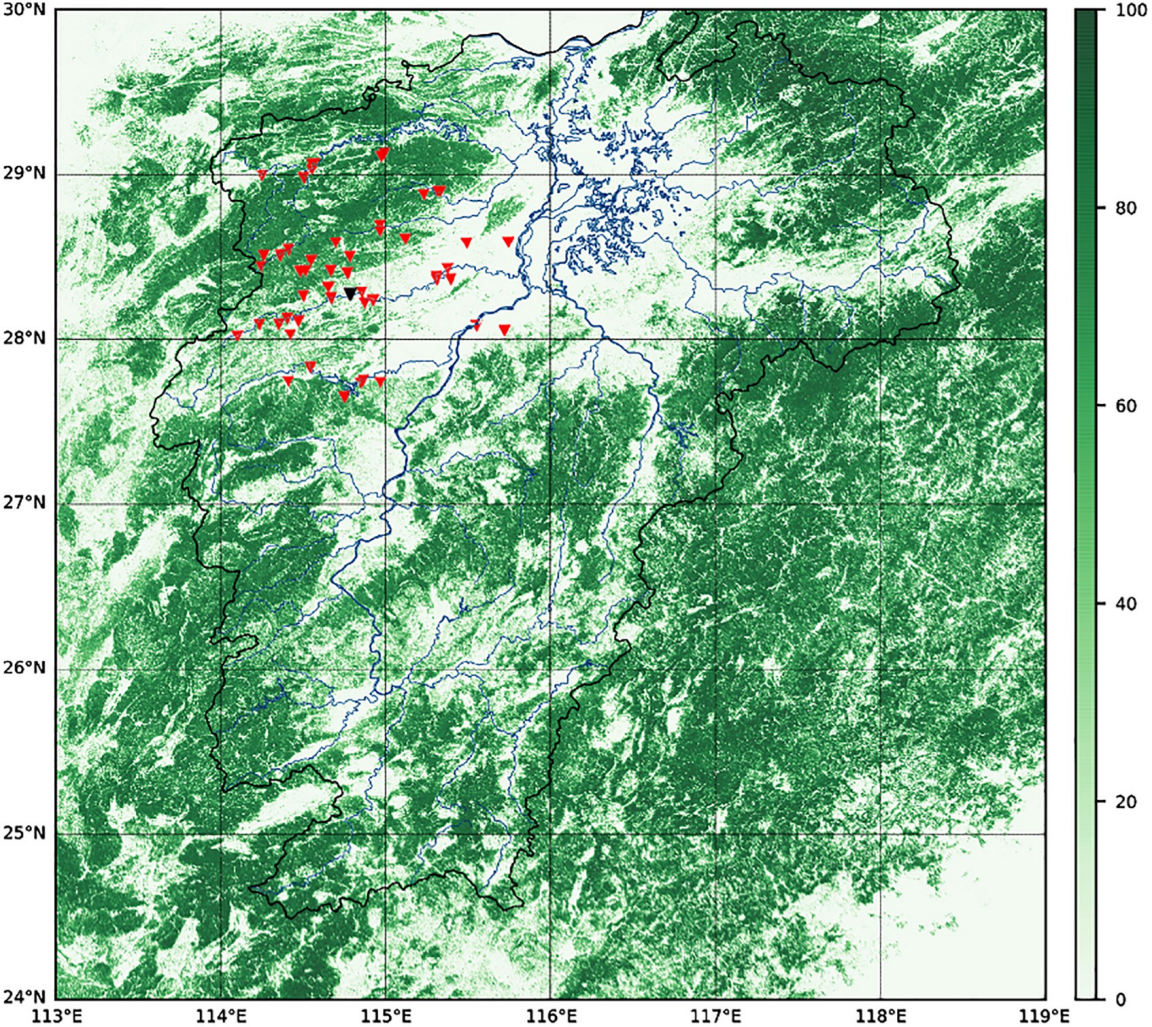
Vegetation coverage over the surrounding area of Jiangxi Province. (The red triangles refer to all stations within 100km from the target basin (indicated as the black triangle).) (The figure was generated by Python with basemap toolkit. The map source and license are accessible from https://github.com/matplotlib/basemap.).

The vegetation coverage is shown in Fig 12. Vegetation coverage and specific geographic attributes such as watershed area and length are shown in Table 3.

**Table 3:** Watershed area, watershed length and vegetation coverage of each station.

Station	Shang Gao	Zha Jin	Jin Xi	Tong Gu
Watershed Area(*km*^2^)	4106.83	664.50	16.64	251.17
Watershed Length(*km*)	126.93	47.58	6.15	23.92
Vegetation Coverage(%)	36.01	37.68	57.79	58.76
Slope	0.0078	0.0253	0.0781	0.0435
Station	Lao Zhuang	Xian Feng	Bai Zhushan	Wan Zhuang
Watershed Area(*km*^2^)	664.50	1730.93	431.33	290.48
Watershed Length(*km*)	34.65	102.39	36.77	37.86
Vegetation Coverage(%)	58.39	61.72	59.20	59.39
Slope	0.0251	0.0121	0.0261	0.0385
Station	Huang Shan	Shen Shan	Jin Ping	Yang Hu
Watershed Area(*km*^2^)	332.39	47.24	301.99	16.62
Watershed Length(*km*)	30.97	12.07	30.12	5.33
Vegetation Coverage(%)	25.19	31.04	62.07	64.79
Slope	0.0162	0.0190	0.0343	0.1112
Station	Xiang Bi	Zhou Shang	Xiu Shui	Deng Bu
Watershed Area(*km*^2^)	32.57	18.14	4647.03	26.44
Watershed Length(*km*)	8.65	4.63	111.80	8.34
Vegetation Coverage(%)	24.98	0.48	57.91	0.06
Slope	0.0351	0.0073	0.0112	0.0023
Station	Yi Feng	Niu Toushan	Zhi Yuan	Shi Shi
Watershed Area(*km*^2^)	17.41	2732.16	52.98	2886.01
Watershed Length(*km*)	6.49	94.70	13.26	108.65
Vegetation Coverage(%)	28.79	38.58	63.94	37.24
Slope	0.0166	0.0103	0.0549	0.0090
Station	Tu Ku	Ling Jiang	Guan Zhou	Teng Qiao
Watershed Area(*km*^2^)	166.16	743.65	516.12	315.97
Watershed Length(*km*)	26.40	56.82	58.27	23.28
Vegetation Coverage(%)	49.68	41.14	65.41	50.41
Slope	0.0564	0.0173	0.0262	0.0404

### 5.2 Comparative model and parameter setting

In this experiment, common prediction evaluation indexes and flood prediction evaluation indexes are selected, including the Root Mean Square Error, Certainty Coefficient, Kling-Gupta Efficiency Coefficient, Nash-Sutcliffe Efficiency Coefficient and Peak Flow Error index. The experiment first compares the model results of different selected source watershed and auxiliary watershed training, and then compares the model results of adjustment training under different views. The specific model is described as follows:

**Model based on geometric similarity (Trans)**: Geometric similarity graph is used to select source basin and auxiliary basin data without optimization and adjustment of multiple spatiotemporal views.

**Model based on geometric similarity and spatiotemporal views (ST-Trans)**: Refer to the model of the paper [48] and adjust the model using the Tri-training concept. The geometric similarity relation graph is used to select the source basin and auxiliary basin data, and the model is optimized and adjusted by multiple spatiotemporal views.

**Model based on spatial similarity (H-Trans)**: The source basin and auxiliary basin data are comprehensively selected using multiple relational maps in spatial similarity without optimization and adjustment of numerous spatiotemporal views.

**Model based on spatial similarity and time view (TH-Trans)**: The source basin and auxiliary basin data are comprehensively selected by using multiple relational graphs in spatial similarity, and the time series data are added for adjustment and optimization.

**Model based on spatiotemporal view adjustment (CoH-Trans)**: Refer to the model of the paper [36] and adjust the model using the Co-training concept. the source basin and auxiliary basin data are comprehensively selected by using the multi relational graph in spatial similarity, and the time series feature and spatial feature are added to adjust and optimize based on the collaborative training method.

**Model based on multiple spatiotemporal views adjustment (STH-Trans)**: the source basin and auxiliary basin data are comprehensively selected by using multiple relational maps in spatial similarity, and the optimal adjustment and optimization are carried out based on the multiple spatiotemporal views proposed in this paper.

Experimental environment: operating system Windows 10, processor Inter i7-9700, 2.4 GHz, memory 32 GB, GPU GTX 1050 Ti.

**Fig 13:**
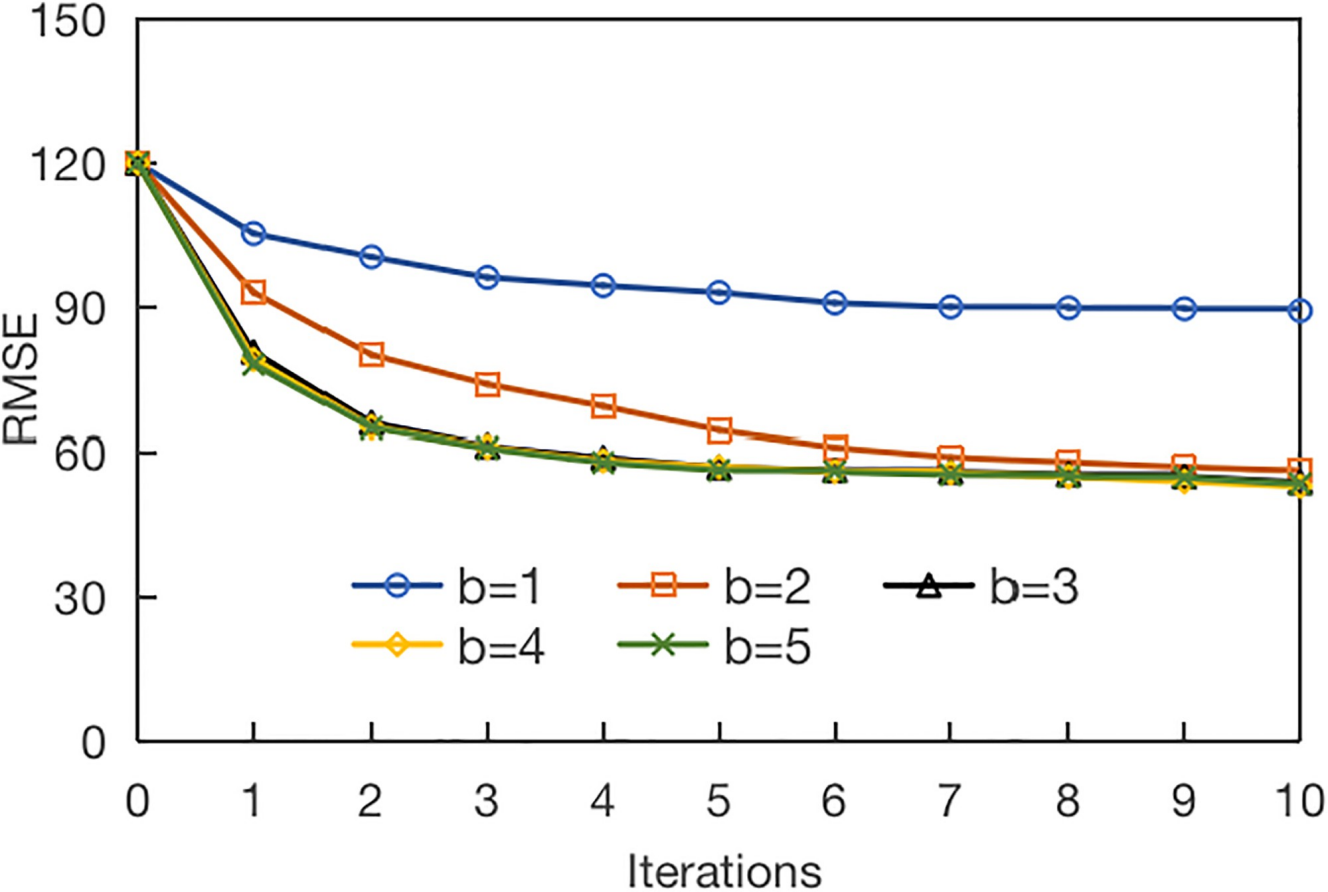
Influence of parameter *b* in the model.

Experimental parameters: the base learner is *M*5 *model tree*, the average weight is set for the initial sample weight, and the maximum number of iterations is 50. To ensure the model’s effectiveness, the parameters of *a* and *b* must be set through training. The experimental setting is *a* = 3 and *b* is [1, 5]. When *b* is 1, the unlabeled sample is not divided. The experimental results are shown in Fig 13.

It can be seen that *RMSE* is decreasing no matter what the value of *b* is, especially the previous iterations. This also indirectly proves the effectiveness of our proposed model. When *b* = 1, the *RMSE* of the model is declining but does not reach the optimal performance. When *b* = 2, the model still needs several iterations to achieve better results. When *b* reaches 3, the model can quickly achieve the optimal effect, so the experimental setting is *b* = 3.

**Fig 14:**
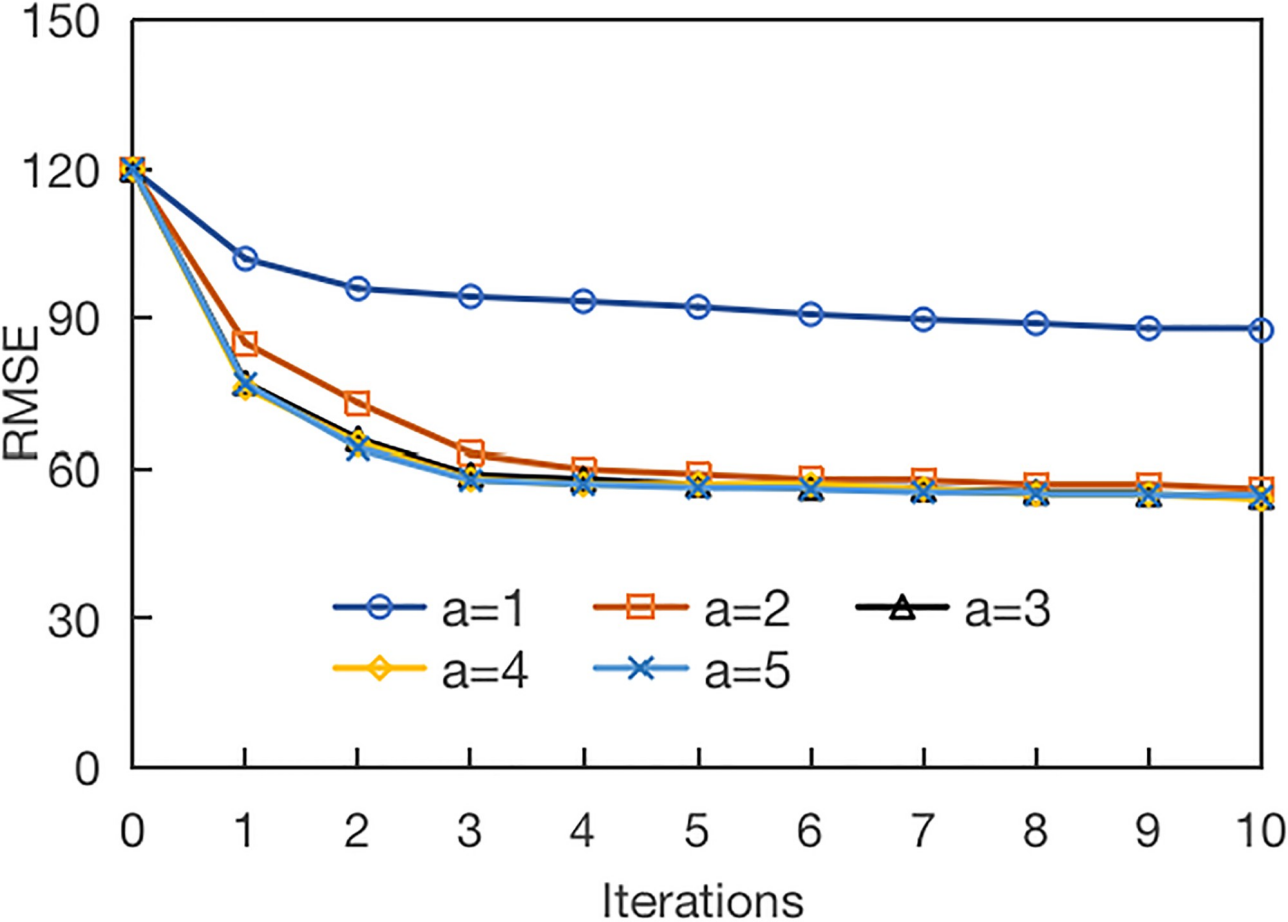
Influence of parameter *a* in the model.

We continue to find the optimal parameters of *a*. The experimental setting is *b* = 3, *a* is [1, 5]. The results are shown in Fig 14.

It can be seen from the figure that the maximum convergence cannot be achieved when *a* = 1. In other cases, the model can reach the maximum convergence after *a* period of time, and obtain better results. In addition, the performance when *a* is taken as 2 is slightly worse than when *a* is taken as 3. After *a* reaches 3, the model performance is similar, and the optimal effect can be achieved quickly, so the experimental setting is *a* = 3.

### 5.3 Experimental results and comparative analysis

**Fig 15:**
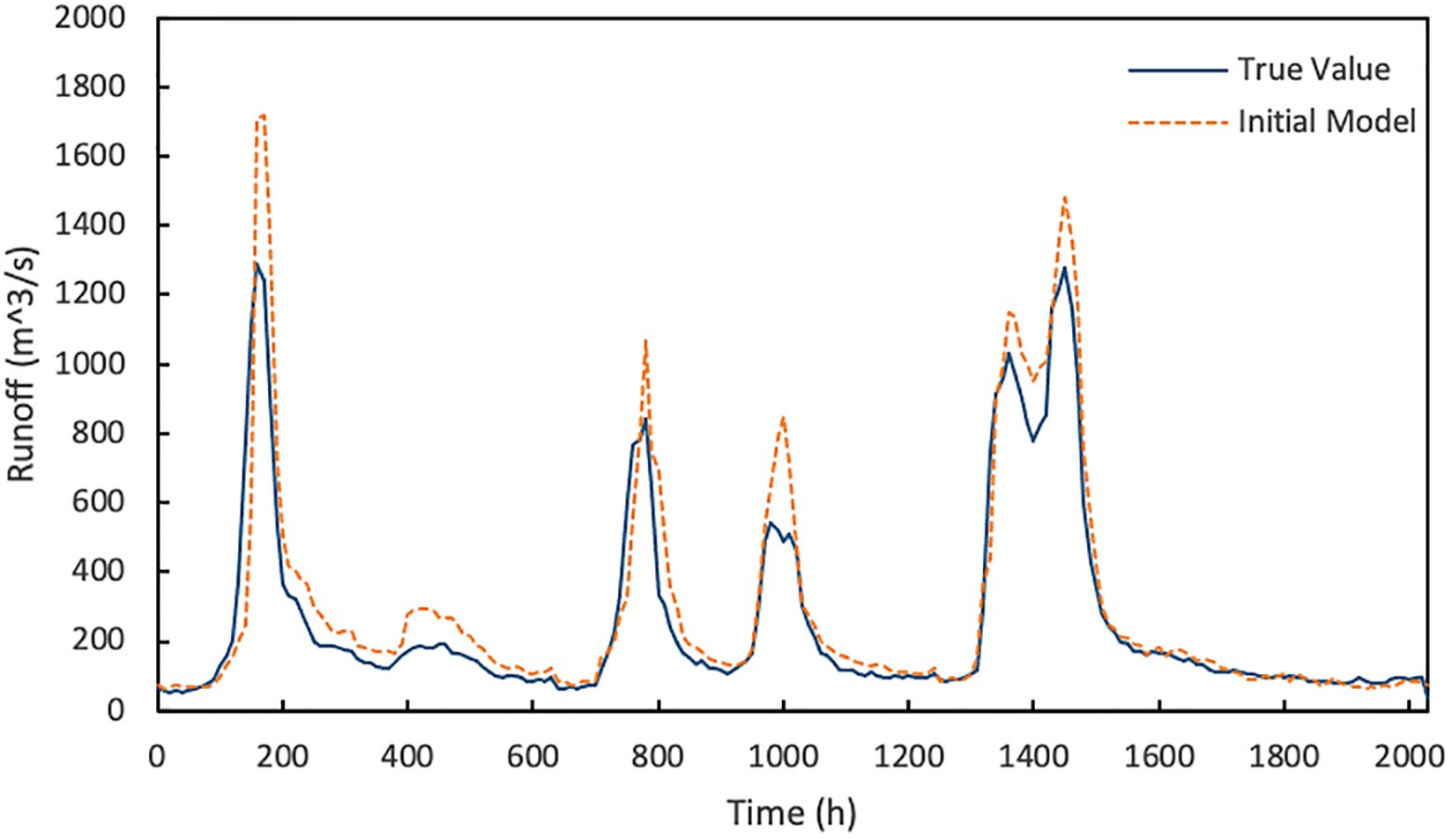
Initial model prediction results.

The experiment establishes an initial prediction model, and the prediction results are shown in Fig 15. It can be seen that the runoff change trend predicted by the initial model is basically consistent with the observations, rising before the flood peak arrives and falling after the peak. The purpose of hydrological data prediction is to predict the coming flood and to conduct water resources scheduling. The initial model achieves this goal, but the accuracy still needs to be improved. The high flood peak prediction value here may also be due to the higher runoff of the auxiliary basin and the surrounding source basin. It can also be found from the comparison of figure above that the upper runoff is generally higher than that of Shi Shi, which may lead to the higher predicted runoff.

In physical process, geometric similarity is the premise of dynamic similarity and kinematic similarity. We selected the source basin and auxiliary basin based on geometric similarity for comparative experiments.

**Fig 16:**
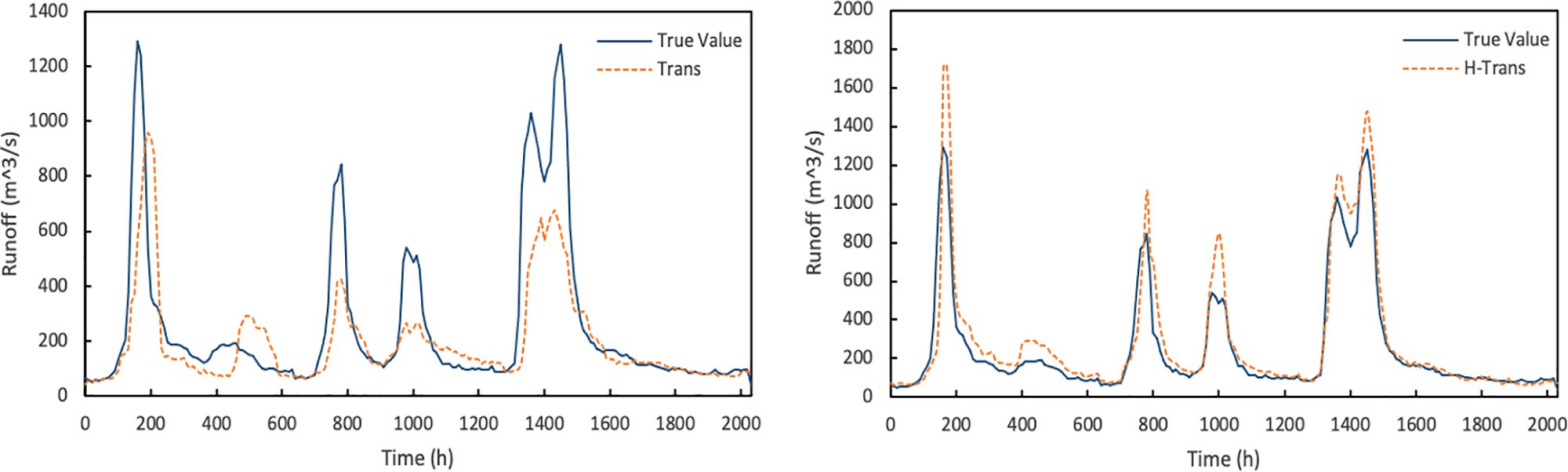
Results of Trans and H-Trans model.

It can be seen from the figure that different dataset selections have an impact on the model results. The Trans model in the figure uses the source basin and auxiliary basin datasets selected based on geometric similarity. The auxiliary basin is Niu Toushan, and the geometric distance between Niu Toushan and Shi Shi is the smallest, their horizontal distance is also small. The H-Trans model uses the data of the source basin and the auxiliary basin selected by the comprehensive factors such as geometric similarity, topographic index and geographical location relationship. The auxiliary drainage basin is Shang Gao. Because the auxiliary basin selected by the Trans model is not as good as that selected by the H-Trans Model selection, Niu Toushan is not the best auxiliary basin, so the model effect trained by the source basin and auxiliary basin selected by the Trans model is worse than that of H-Trans model. Fig 16 shows that the training data selected by the method proposed in this paper has better training results and can predict higher flood peaks. The hydrological prediction in the ungauged areas is for flood prediction. The prediction of peak flow is important in flood warning and emergency rescue. Therefore, the selection of appropriate datasets for the model is crucial.

**Fig 17:**
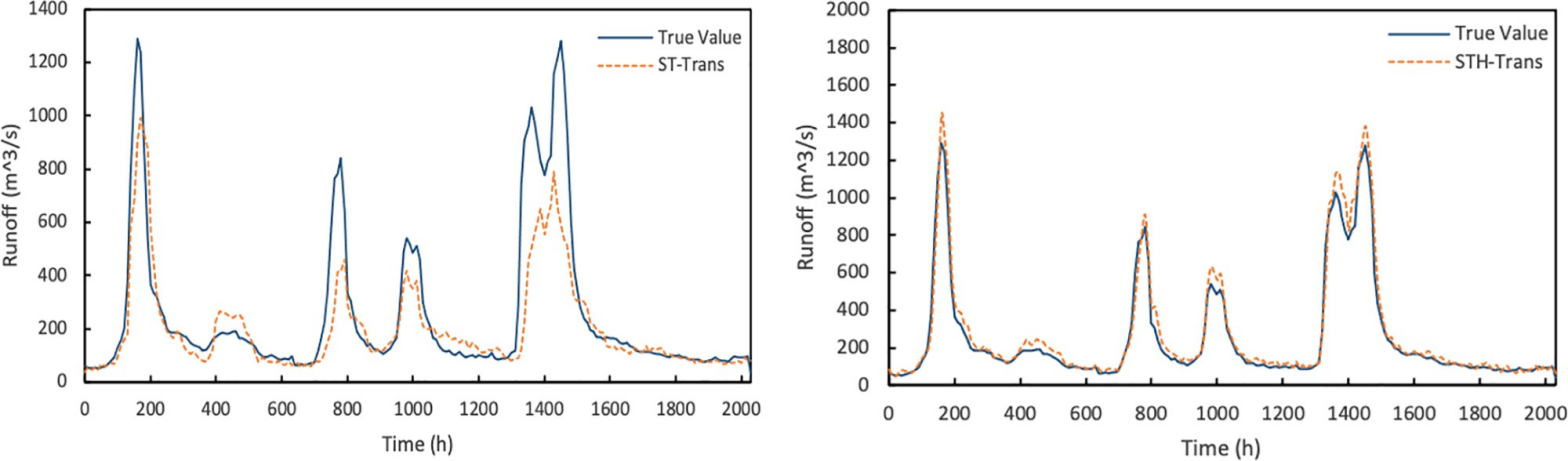
Results of ST-Trans model and STH-Trans model.

Then we train the models based on multi-spatiotemporal views adjustment for the different training data of the source basin and auxiliary basin. The experimental results are as follows (Fig 17):

It can be seen in the figure that the training dataset selected by the method proposed in this paper can get better results. Although the training results of the source basin and auxiliary basin data sets based on geometric similarity are better than those before multi-view adjustment, the prediction of flood peak still has problem. At the same time, the model adjusted by multiple spatiotemporal views is better than the general transfer learning model, and its accuracy is significantly improved. The STH-Trans model not only incorporates spatial features, but also has been improved through multiple spatiotemporal views.

**Fig 18:**
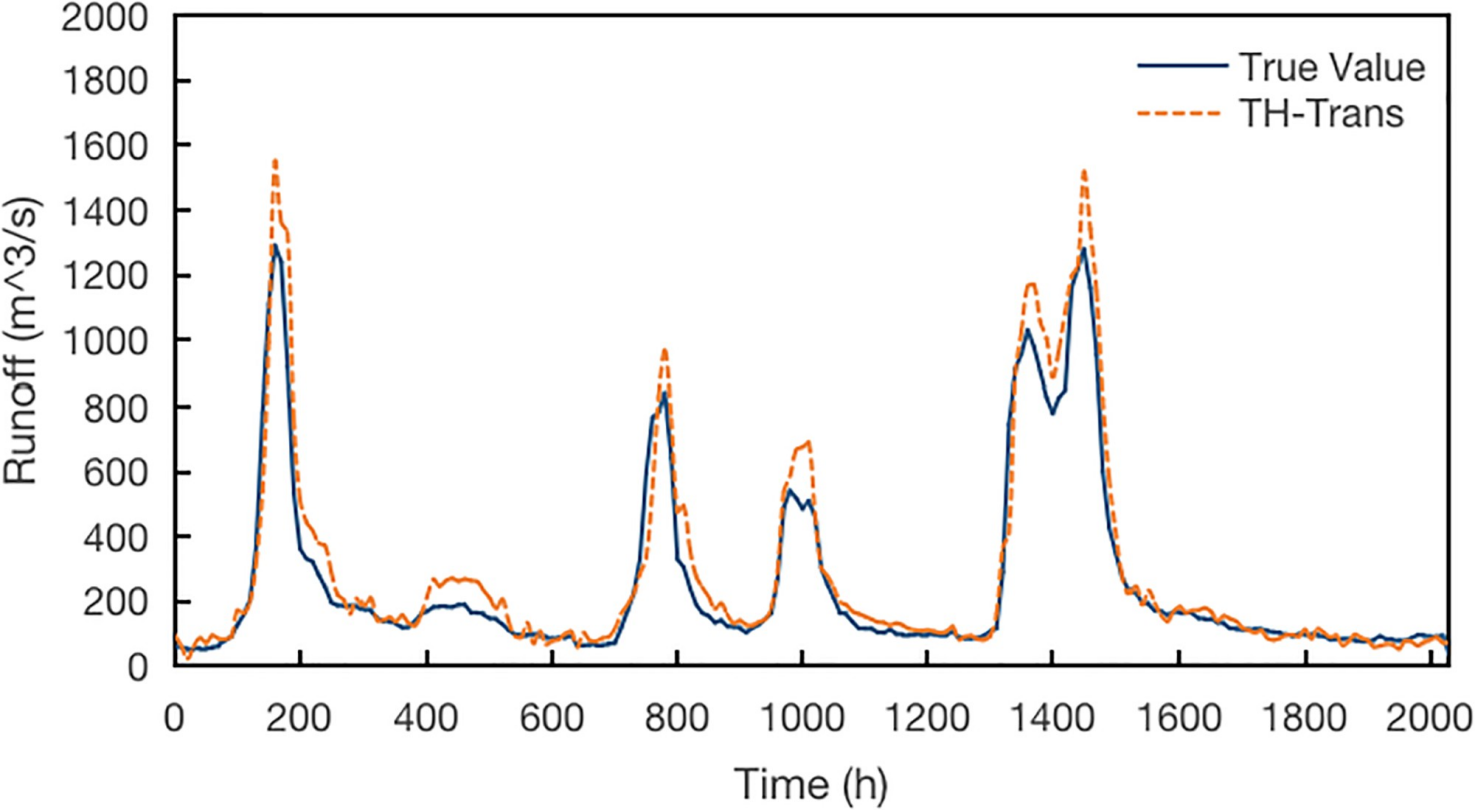
Model results with temporal features.

The hydrological prediction models under different views are compared. In the hydrological process, rainfall has a significant influence. The experiment first adds rainfall to train the model, adjusting the initial model based on a single time series feature. The results are shown in Fig 18:

It is obvious from the figure that the TH-Trans model with time series feature adjustment is more accurate than the initial H-Trans model. Although the geographical position in the spatial view and the topography and geomorphology in the attribute view have inextricably linked relations with the formation of hydrological phenomena, there is no direct causal relationship, so the spatial view and attribute view are not separately trained and compared.

**Fig 19:**
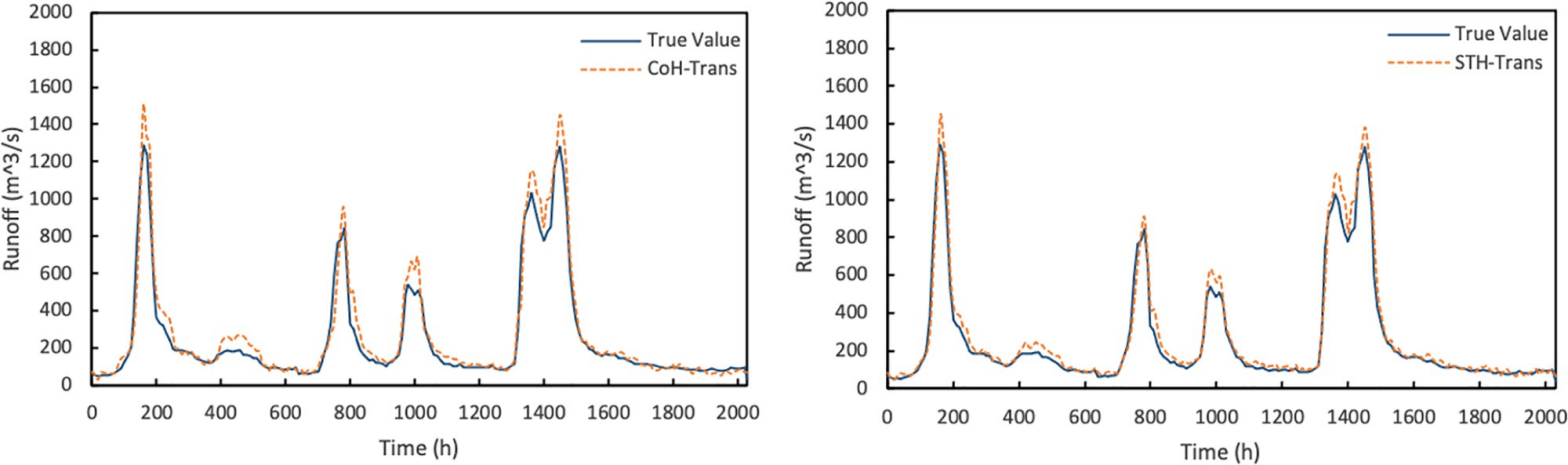
Results of CoH-Trans model and STH-Trans model.

Next, compare the models based on different views. The experimental results are shown in Fig 19. The model based on dual spatiotemporal view is adjusted based on the time series view and spatial geographical location view, and the model is trained using the collaborative training method.

It can be seen from the figure that the results of the CoH-Trans model based on the dual spatiotemporal view are better than those of the H-Trans model, while the STH-Trans model based on the multi spatiotemporal view adds an attribute view on the basis of the CoH-Trans model, which fits better and makes the local prediction of the flood peak more accurate.

Table 4 compares the predicted results of the migration model for the above models. It can be seen that the ST-Trans model and the STH-Trans model adjusted by multiple spatiotemporal views are much better than the original model without adjustment such as Trans and H-Trans, with smaller root mean square error and higher accuracy. The auxiliary basin and source basin selected in this method can apparently help hydrological migration. The STH-Trans model based on multiple perspectives has better NSE and KGE, higher *R*^2^ and lower RMSE than TH-Trans and CoH-Trans. This may be because the STH-Trans model considers factors such as geographical location and terrain, making predictions more accurate.

**Table 4:** Comparison of various transfer models from different perspectives.

Evaluation Index	Coefficient of Certainty (*R*^2^)	Root Mean Square Error (RMSE)	Nash-Sutcliffe Efficiency Coefficient (NSE)	Kling-Gupta Efficiency Coefficient (KGE)
Different Datasets				
Trans	0.601	193.641	0.423	0.501
H-Trans	0.904	112.932	0.475	0.565
ST-Trans [48]	0.780	159.747	0.649	0.773
TH-Trans	0.953	77.117	0.788	0.824
CoH-Trans [36]	0.962	62.695	0.956	0.843
STH-Trans	0.984	50.114	0.978	0.885

One of the purposes of hydrological forecasting is to predict floods, so it is to be able to predict the arrival time and peak of floods. In addition to comparing hydrological prediction results, we also need to compare and predict extreme flood events that occur in hydrological prediction, especially the accuracy of flood peak prediction. The flooding process with the highest peak value in the prediction sequence is selected for comparison, as shown in Table 5.

**Table 5:** Comparison of transfer model results of one flood process.

Evaluation Index	Coefficient of Certainty (*R*^2^)	Root Mean Square Error (RMSE)	Peak Error (PE)
Different Datasets			
Trans	0.328	491.932	-332
H-Trans	0.649	356.661	430
ST-Trans [48]	0.615	253.046	-296
TH-Trans	0.854	181.671	210
CoH-Trans [36]	0.870	176.296	191
STH-Trans	0.949	115.442	152

It can be seen from the table that the flood peak value predicted by the dataset selected by the geometric similarity ST-Trans model does not go up, and the selection method of the source basin and auxiliary basin in this paper can better predict the peak value.

The adjusted model performs better than the initial model, with higher precision, lower root mean square error and smaller peak error. In the adjusted model, the precision of the CoH-Trans model based on a dual spatiotemporal view is improved, and the error is reduced compared with the TH-Trans model based on a single view of time series. Among them, the STH-Trans model has the best effect. It not only improves the accuracy by 30% compared with the H-Trans model, 9.5% compared with the TH-Trans model, and 7.9% compared with the CoH-Trans model, but also dramatically reduces the root mean square error. In terms of flood peak error, the STH-Trans model under the multi temporal and spatial view also performs best, with the minimum flood peak error. It can be seen from the figure that the H-Trans initial model and TH-Trans model lag in peak time, while the STH-Trans model can accurately predict or even advance. Flood forecast is to give early warning of floods, and the prediction of peak value in advance can better leave preparation time for flood control and risk resistance. On the basis of considering spatial features, compared to single view and dual spatiotemporal view models, the STH Trans model not only considers geographical location, but also geographical attribute factors, including some terrain factors, which makes predictions more accurate.

## 6 Conclusion

This paper proposes the STH-Trans model for small and medium-sized ungauged basins which have geographic terrain data. The model integrates transfer learning and semi-supervised learning which taken into account the spatial characteristics of the target basin and the spatiotemporal characteristics of surrounding basins. The innovation and advantages of this article are as follows.

(1) The STH-Trans model employs Transfer learning to initiate the results by training the base dataset with the largest similarity. Compared to parameter transplantation in neighboring areas, this method compares the geographic terrain of the watershed and finds the most suitable migration watershed.
(2) The STH-Trans model refines and improves the experimental results based on geographic characteristics, basin specific characteristics, surrounding watershed characteristics, and rainfall, improving the accuracy of model prediction.
(3) The STH-Trans model reduces the dependence on expert knowledge and addresses data gaps often encountered in traditional data-driven models. This results in enhanced prediction accuracy and a more versatile application across different scenarios.

Moreover, most of the ungauged basins are remote and backward areas, and our research results can provide flow prediction for these basins. In addition, it can not only provide data support for water resource management, but also provide decision-making support for flood prevention and control.

However, the model in this paper still has some limitations.

(1) The watershed tested in this model has a relatively flat terrain and is located in a plain area, belonging to a subtropical humid climate. Therefore, the effectiveness of the model in arid areas still needs to be considered.
(2) Although the model in this article is aimed at ungauged basins, it still requires a large amount of other data, including geographic terrain data, rainfall data, as well as hydrological and terrain data of surrounding watersheds.
(3) The model didn’t deepen the analysis to the mechanisms behind the hydrological function. It focuses more on data analysis rather than physical processes, and its interpretability is weak. This is also a common problem with machine learning models. However, process understanding is necessary while using machine learning-based models for hydrologic predictions [49, 50].

In the future, in terms of models, we will consider incorporating temporal similarity and constructing hydrological spatiotemporal similarity to further refine domain selection and improve prediction accuracy. In terms of application, we consider conducting research on semi-arid and semi-humid regions as well as arid regions. At the same time, we expect to conduct interpretability analysis on machine learning models by combining geographical terrain and hydrological and physical mechanisms, in order to improve the interpretability of the model.
